# Development of a Semi-Automated Tool Based on Computer Vision Methods for Safety Assessment of Micromobility Users

**DOI:** 10.3390/s26185713

**Published:** 2026-09-09

**Authors:** Alejandra Sofía Fonseca-Cabrera, David Llopis-Castelló, Alfredo García

**Affiliations:** Highway Engineering Research Group, Universitat Politècnica de València, 46022 Valencia, Spain; dallocas@upv.es (D.L.-C.); agarciag@tra.upv.es (A.G.)

**Keywords:** micromobility, computer vision, video processing, lateral position, speed estimation, bike lane, road safety, infrastructure assessment, traffic monitoring

## Abstract

**Highlights:**

**What are the main findings?**

- A semi-automated computer-vision tool extracts the lateral position and instantaneous speed of micromobility users from a single bird’s-eye-view camera recording.
- Validation in controlled tangent and curved sections showed negligible bias and deviations within practical tolerances.

**What are the implications of the main findings?**

- Lane positioning and speed can be jointly monitored at specific locations of cycling infrastructure without instrumented vehicles or trained detection models.
- The tool reduces processing time compared with manual video analysis and eliminates operator subjectivity in the tracking process.

**Abstract:**

The operational behavior of micromobility users is a key indicator of the safety performance and design quality of cycling infrastructure; yet, existing video-based methods either require intensive manual processing or locate users coarsely through the centroid of the bounding box. This study presents and validates a semi-automated computer-vision tool that extracts the lateral position and instantaneous speed of micromobility users from bird’s-eye-view video recordings acquired with a single camera. The tool, implemented in Python 3.10.11, integrates bike lane segmentation, background-subtraction-based detection, multi-object tracking, and a heatmap-based contour extraction that places the measurement point at the wheel–pavement contact, providing a physically meaningful reference at predefined control sections. Validation was conducted in controlled tangent and curved sections, against physical distance references and previously verified e-scooter speed readings. In the tangent section, lateral position estimates showed a negligible bias, with a mean error below 1 cm and 99% of observations within ±5.0 cm, while over 70% of speed estimates were within ±2.0 km/h tolerance. In the curved section, the tool slightly underestimated lateral position and overestimated speed, with errors remaining within the practical tolerances. These results support the use of the tool for operational and safety studies of micromobility infrastructure under controlled conditions, reducing processing time without requiring trained detection models.

## 1. Introduction

Micromobility has expanded rapidly in urban areas over the last decade, with bicycles and e-scooters, both private and shared, becoming established components of urban transport systems [1,2] as alternatives to motorized individual transport.

Nowadays, cycling is widely promoted as an active transport mode for first- and last-mile trips, as reflected by the widespread implementation of public and private bike-sharing systems in cities worldwide. In recent years, e-scooters have also emerged as an alternative for covering the same mobility gap, with particularly rapid growth in the United States in 2018 [3] and subsequent expansion to many cities worldwide. Their popularity has been strongly driven by shared mobility systems, although this growth has also raised issues such as irresponsible riding, improper parking, sidewalk clutter, and vandalism [4].

In general, promoting micromobility through the effective integration of bicycles and e-scooters into the urban transport system remains a complex challenge that has generated regulatory and operational conflicts for stakeholders [5]. Additionally, the shared usage of dedicated cycling infrastructure by both bicycles and scooters has proven challenging due to their different operating characteristics, particularly in terms of speed and maneuverability.

For this reason, this growth of micromobility usage has been accompanied by increasing safety concerns, reflected in the rising number of crashes and injuries involving micromobility users [6,7]. In this context, the operational behavior of cyclists and e-scooter riders constitutes a critical indicator for understanding both the safety performance and the design quality of urban cycling infrastructure.

A wide range of approaches has been employed to evaluate micromobility user behavior. Commonly analyzed variables include speed, lateral clearance, and interactions [8]. For a more in-depth assessment of operational behavior, the most relevant variables are speed and trajectory, as they allow for identifying how users operate under different infrastructure conditions [9]. The lateral position of users is particularly meaningful, since it relates to the effective width required by the infrastructure and to the clearance available in passing and meeting maneuvers [10,11]. Moreover, micromobility systems encompass diverse scenarios, including segregated infrastructure, shared roadways, and experimental tracks, and different methodological approaches have been developed to capture and analyze user behavior across these contexts.

Traditional studies have largely relied on manual observation or sensor-based measurements. In this regard, the instrumentation of micromobility vehicles has emerged as an increasingly adopted method for in-field data collection, enabling naturalistic data acquisition. Fonseca-Cabrera et al. [12] used an instrumented e-scooter equipped with an ultrasonic sensor and a low-cost data acquisition system [13] to measure lateral clearance between users across different bike lane typologies, while Shoman et al. [14] conducted measurements on three types of facilities using an instrumented bicycle fitted with an Inertial Measurement Unit (IMU), GPS, and laser scanner. Similarly, Schepers et al. [11] employed an instrumented bicycle with LiDAR sensors to measure the lateral position of cyclists under different bike lane width conditions. Despite their accuracy and richness of data, these methods present limitations in terms of scalability and deployment over large study areas.

In recent years, digital image processing has become an increasingly prevalent tool for analyzing micromobility behavior. Video recordings can be considered a naturalistic data collection method, as users are generally unaware of the presence of cameras and therefore do not alter their behavior; naturalistic video data have been used, for instance, to model the interactions between cyclists and motorized vehicles at unsignalized intersections [15]. Moreover, a wide range of variables can be extracted and analyzed from video data, making it a highly versatile strategy. Nevertheless, it typically requires substantial effort for data filtering and processing. Video image processing can be performed using different tools and techniques. One commonly used software for extracting speed and trajectories is Kinovea. Sanjurjo-de-Nó et al. [16] processed a set of recorded videos using this motion analysis software to investigate behavioral differences between men and women, considering speed as a surrogate measure of user behavior on bike lanes. Data obtained using Kinovea can be considered reliable because the software applies its own calibration based on a ground-truth measurement. However, the process still involves operator subjectivity during manual tracking and is time-consuming. In this study, Kinovea was used to manually measure users’ lateral position within the lane and to estimate speed by tracking their movement between two predefined markers, allowing speed to be calculated from the known distance and corresponding travel time.

More recently, the automation of video processing has gained significant attention. Computer vision techniques enable automated user tracking and the extraction of operational parameters, such as speed, trajectory, and maneuver dynamics, from video recordings, facilitating the analysis of large datasets and the identification of behavioral patterns associated with potentially risky maneuvers [17]. Applications include the automatic detection of critical traffic situations involving cyclists, such as the system developed by Detzer et al. [18], which automatically detects atypical and potentially dangerous traffic situations involving interactions between cyclists and other road users, based on trajectory extraction and a trained artificial neural network. The trajectories were validated using high-precision Trimble GPS data. However, occlusions and shadows can alter the detected center of gravity and consequently affect the reconstructed trajectory. The study also acknowledges the difficulty of tracking the wheels against the background, while occlusions can interrupt cyclist monitoring. In addition, the method still requires post-processing and manual evaluation.

At a larger scale, characterization of cycling near misses has also been addressed using machine intelligence [19]. The proposed framework automatically analyzes video streams to detect and classify near-miss events by considering interactions among cyclists, other transport modes, and the built environment. The approach combines computer vision and tracking techniques, including YOLOv5 with COCO-pretrained weights and DeepSORT, with a dedicated CyclingNet model for action recognition.

Other applications include the generation of multimodal trajectory datasets from drone recordings with a bird’s-eye view at a height of 110 m [20]. In general, many of these developments rely on supervised object detection models and bounding box estimation [21], even to determine helmet usage [22], which requires dedicated training and the preparation of annotated datasets.

Regarding the estimation of user position, Beitel et al. [23] analyzed pedestrian–cyclist interactions by extracting user trajectories from videos recorded with a fixed camera placed at a height of 6 m, using the computer vision software Traffic Intelligence, an open-source object tracking tool. In their approach, trajectories were derived from the centroid of the bounding box associated with each user. Similarly, Tolosue et al. [24] developed a computer vision tool to estimate lateral passing distance and overtaking speed of motorized vehicles using the same computer vision software, which performs road-user detection, tracking, classification, passing-distance extraction, and speed estimation. This method is suitable for interaction and conflict analysis; however, it does not provide precise information on the ground-level positioning of micromobility users.

In contrast, Ronné et al. [25] developed an automated method to quantify the path-following behavior of cyclists in an experimental circuit. In this case, the trajectory was directly estimated by measuring the distance between the center of the front wheel and a predefined reference line, allowing for a more precise representation of the cyclist’s position relative to the infrastructure. Video data were collected using a camera mounted on the bicycle handlebar, within a controlled experimental setup involving volunteer participants.

Automated methodologies have thus been successfully applied in both naturalistic and experimental studies, yielding robust and reliable results. However, existing automated approaches either locate users coarsely through the centroid of the bounding box or estimate precise positions only for specific tasks and instrumented setups. An automated method capable of measuring the actual position of micromobility vehicles relative to the infrastructure and calculating the speed at the same location from standard video recordings is still lacking.

Building on these developments, the objective of this study is to develop and validate a semi-automated tool to extract the lateral position and instantaneous speed of micromobility users along bicycle infrastructure segments, including tangent and horizontal curve sections. Unlike previous approaches, the proposed method establishes a new reference for trajectory estimation by defining the user’s position based on the wheel–ground contact point, rather than the centroid of the user’s bounding box, thereby providing a more accurate representation of the user’s actual location on the infrastructure. The proposed framework integrates geometric reference definition, motion analysis, and tracking algorithms to ensure precise and consistent data extraction from bird’s-eye-view video recordings. The tool was validated under controlled experimental conditions in both tangent and curved sections, comparing its lateral position and speed estimates against ground truth references in order to assess its measurement accuracy.

## 2. Methodology

The methodology is organized into two main parts. First, the development of the proposed tool is described, including the input requirements, software environment, processing stages, and final outputs generated. This includes bike lane segmentation, user detection and tracking, path contour extraction, and the quantification of lateral position and speed at predefined control points.

Second, the validation methodology is presented, including the experimental sections, participants, data collection, video acquisition parameters, and reference measurements used for lateral position and speed validation. The distance references consisted of a cobblestone mesh in the tangent section and a fixed known distance in the curved section, while speed validation was based on e-scooter speed readings previously verified against distance–time estimates. Finally, the error variables, descriptive statistics, inferential tests, tolerance-based assessment, and agreement analyses used to evaluate tool performance are defined. This methodological structure provides the basis for implementing the tool and assessing its measurement accuracy.

### 2.1. Tool Development

**Figure 1:**
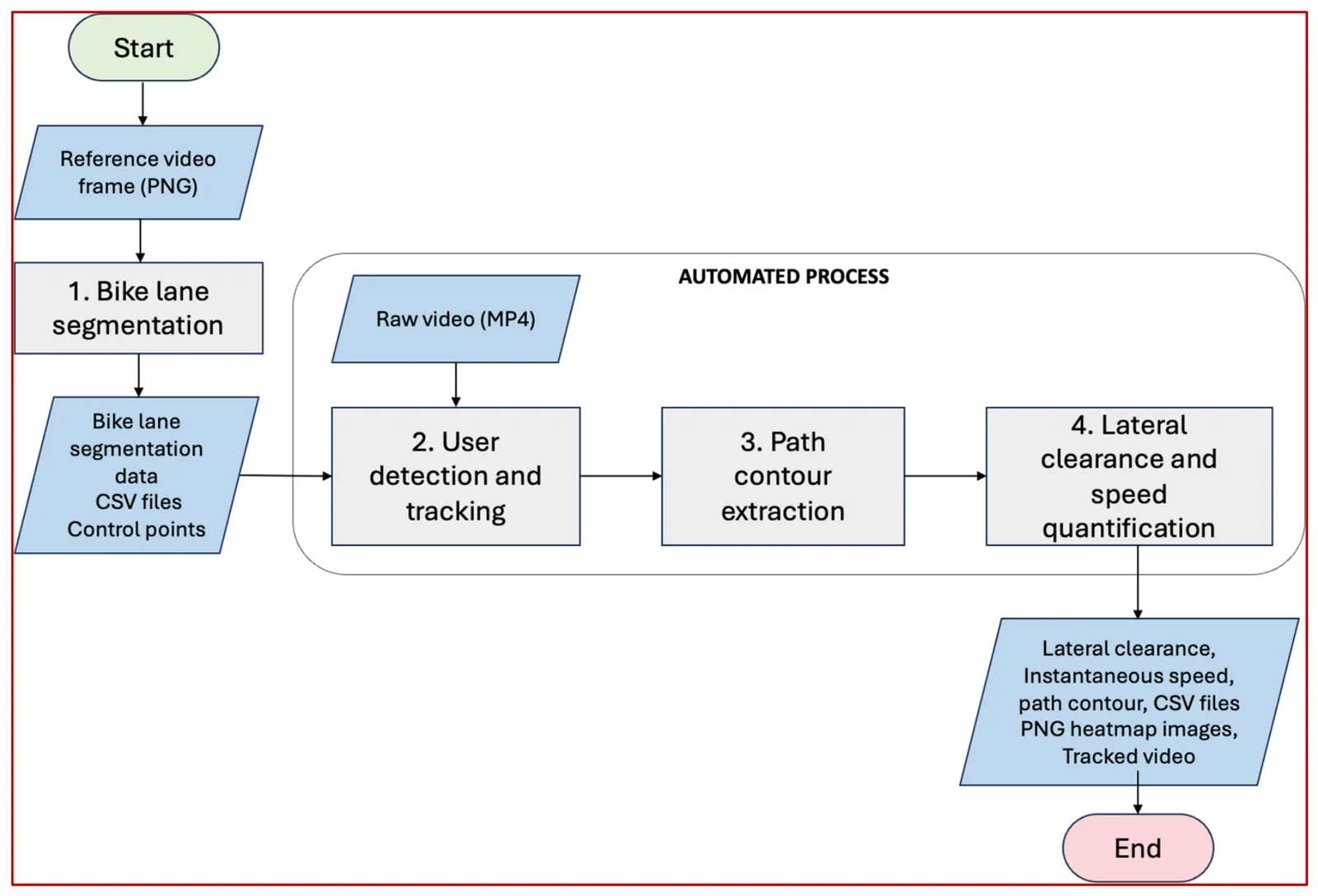
Pipeline procedure.

The proposed methodology estimates user trajectories and instantaneous speed from video sequences through a computer-vision pipeline that integrates foreground segmentation, multi-object detection and tracking, cumulative spatial heatmap generation, contour extraction, and geometric analysis with respect to a predefined lane reference axis and a set of control points. As illustrated in Figure 1, the pipeline is designed to quantify the lateral position of each tracked user trajectory from the lane centerline at selected control points, while simultaneously estimating the corresponding instantaneous speed at those locations.

The method generates both qualitative and quantitative outputs. The qualitative outputs consist of annotated PNG images and tracked videos, whereas the quantitative outputs are stored in CSV files containing the lateral position values with respect to the lane centerline and the estimated speeds. The pipeline was implemented in Python 3.10.11 and is organized according to the processing stages described in the following subsections. The source code is openly available on GitHub and archived in Zenodo (see Data Availability Statement).

The only required input is a bird’s-eye-view video recording of the scene, with a resolution of 1920 × 1080 pixels and a video frame rate of 30 FPS. Video quality and frame rate are fundamental for the reliability of the outputs, since the tool relies on two conversions that link the image domain to real-world magnitudes: a spatial calibration that relates pixels to centimeters through the known lane width, and a temporal reference in which the frame rate represents real time for speed estimation. Consequently, recordings with substantially different resolution or frame rate would require a proportional adjustment of the processing parameters expressed in pixels and frames (e.g., the contour-extraction thresholds and the ±5-frame speed window) in order to maintain the quality of the generated data.

#### 2.1.1. Bike Lane Segmentation

**Figure 2:**
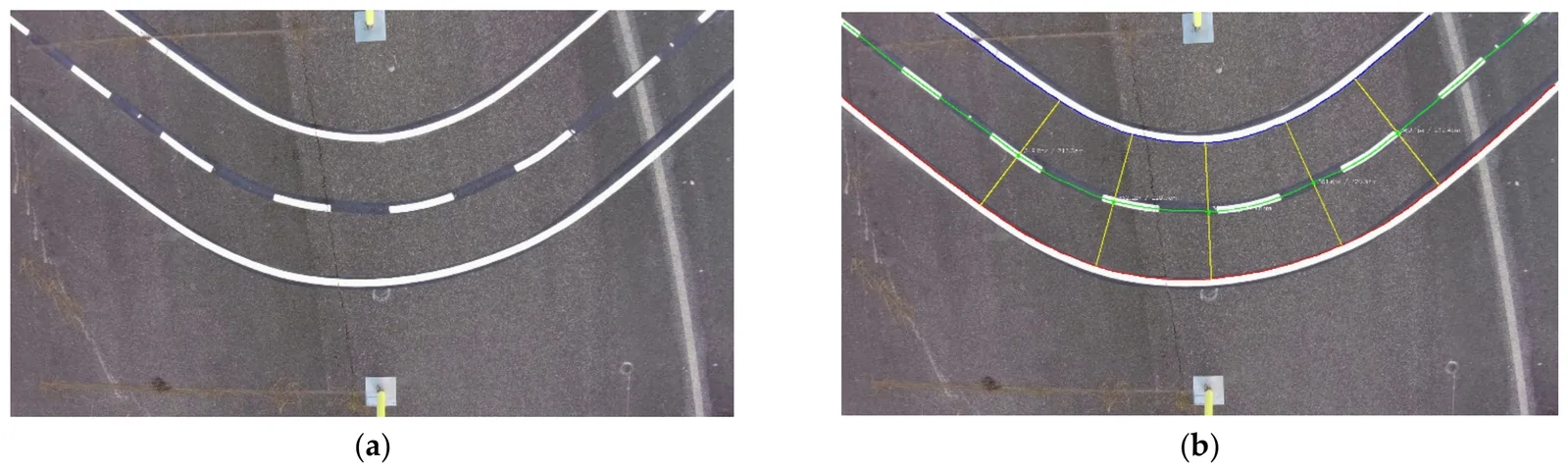
Bike lane segmentation. (**a**) Reference video base frame; (**b**) Reference video base frame segmented for the processing.

The first stage of the proposed pipeline defines the geometric representation of the bike lane, which serves as the spatial reference for the subsequent trajectory, lateral position and instantaneous speed analyses. This procedure is applicable to both curved and tangent lane sections, provided that the video is recorded from a bird’s-eye-view perspective. After the target video sequence is selected, the pipeline extracts a representative reference frame to define the Region of Interest (RoI) (Figure 2a). The lane geometry is then interactively specified within the script by drawing the main geometric components of the lane: (i) the lane centerline, (ii) the left and right lane boundaries, (iii) control points distributed along the centerline, and (iv) a scale reference based on the known physical lane width for pixel-to-metric calibration.

The number and location of control points are user-defined and can be adjusted according to the specific analysis section and the positions at which lateral position and speed need to be computed. In curved sections, they are typically predefined at characteristic locations of the alignment, such as the point of curvature (PC), several intermediate points (INT), the midpoint (MP), and the point of tangency (PT) (Figure 2b). The pipeline is set to assign automatically green color to the center line and the control points defined, blue and red to the edges and yellow to the perpendicular lines for each control point between the edges. This segmentation procedure is transferable to other lane geometries under the same bird’s-eye-view acquisition condition.

This stage produces both visual and numerical outputs. A PNG verification image is generated to assess the consistency of the lane delineation, the transverse control sections, and the scale reference (Figure 2b). In addition, CSV files are created to store the pixel coordinates of the centerline, lane boundaries, and control sections associated with each selected control point.

The resulting geometric files are subsequently used as input references for the video-processing stage.

#### 2.1.2. Detection and Tracking

**Figure 3:**
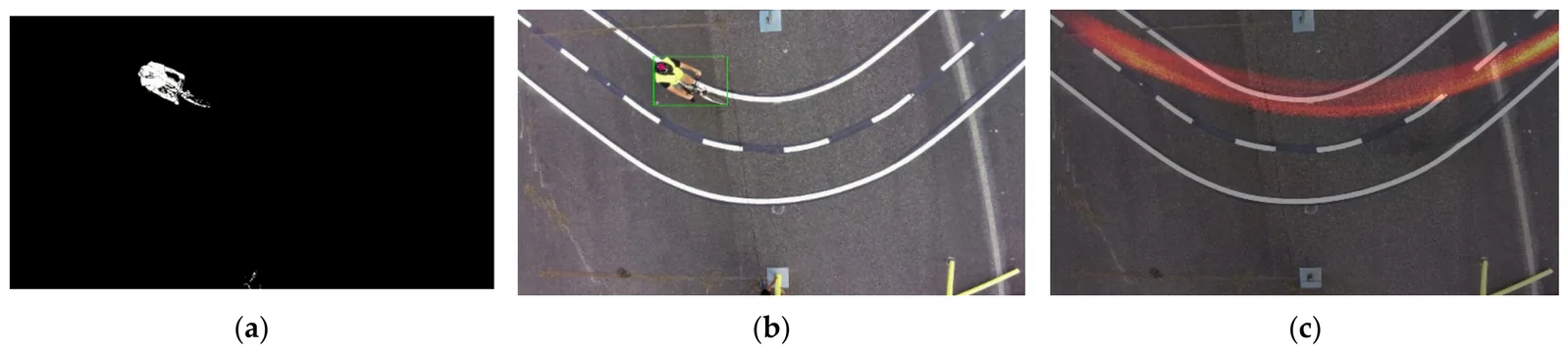
Detection and tracking-heatmap obtention. (**a**) Background subtraction; (**b**) user detection and tracking; (**c**) User heatmap generation.

The video-processing stage starts with the identification of moving users within the scene. For each frame, foreground regions are extracted using a Gaussian Mixture Model (GMM)-based background-subtraction algorithm, implemented through cv2.createBackgroundSubtractorMOG2 in OpenCV 4.10,0 [26]. The resulting foreground mask is binarized using a fixed threshold, and connected moving regions are identified by contour extraction. This intermediate step separates dynamic objects from the static background and provides the candidate detections used by the tracking module (Figure 3a).

The detected foreground regions are subsequently represented as bounding boxes derived from the extracted contours and passed to the DeepSORT tracker. Each confirmed track is assigned a unique identifier and is updated over consecutive frames (Figure 3b). For every tracked object, the current bounding box is retrieved, and the corresponding track ID is used as the user identifier. The foreground mask associated with the object is binarized and then accumulated into an individual user-level heatmap, so that each frame contributes a unit count at every occupied pixel; the resulting heatmap is therefore a map of temporal occupancy, expressed as a number of frames, and retains no photometric information. The bounding-box centroid and frame index are stored to generate the preliminary trajectory of the user.

As a result, each tracked user is characterized by two complementary representations: a space–time trajectory and a cumulative spatial occupancy footprint. The individual heatmaps summarize the areas occupied by each user throughout the video sequence and provide the input for the subsequent trajectory-contour extraction stage (Figure 3c).

#### 2.1.3. Extraction of the Path Contour

To recover the operational trajectory of each user, a path contour is extracted from the individual heatmap by analyzing the spatial concentration of accumulated pixels. The heatmap band is wider than the actual tire track because it incorporates the entire vehicle and rider body. However, the wheels systematically produce a higher pixel concentration than the body and, regardless of the direction of travel, the concentration associated with the wheel–pavement contact is the one lying lowest in the image (largest y-coordinate, with the image origin at the top-left corner). The extraction procedure therefore targets the lower edge of the high-concentration region of the heatmap, while explicitly accounting for the way the bird’s-eye perspective modulates the apparent height of this region along curved sections.

The per-user heatmap is first converted into a band mask through binarization followed by morphological closing and opening (15-pixel and 3-pixel kernels, respectively), retaining only connected components with an area of at least 150 pixels. The raw accumulation counts restricted to this band define a weight field. The lane centerline is then densified at 4-pixel steps to obtain a dense set of measurement stations and, at each station, the transverse direction is taken from the nearest control perpendicular defined during the segmentation stage. The weight field is sampled along each transverse direction over a ±400-pixel range, yielding a transverse accumulation profile for every station.

Three candidate estimates are computed from each accumulation profile. First, a high-concentration mask is defined relative to the local peak of the profile, retaining the positions whose accumulated occupancy reaches at least 60% of the local maximum. The use of a relative threshold, rather than an absolute count, makes the extraction robust to variation in the maximum heatmap response across sections and videos. Among the contiguous high-concentration runs, the one reaching the largest y-coordinate is selected, and its lower edge is taken as the estimate of the wheel footprint. Second, the lower edge of the full band is computed as the absolute lower contour of the occupied region. Third, the occupancy-weighted centroid of the same high-concentration core may be taken as the estimate, placing the contour at the center of mass of the accumulated occupancy rather than at its lower boundary.

Along curves, the apparent position of the contact point within the accumulated band varies between the extremes and the apex; where that variation is significant, the final contour blends both boundaries. The control points are reordered, if necessary, according to their arc-length position along the centerline, and the concavity of the curve is detected automatically from the centerline geometry. In curved sections, these control points correspond to the characteristic locations defined during the segmentation stage, with the extreme control points typically located at the PC and PT of the curve, the central one at the MP, and as many intermediate points (INT) as needed. A cosine weighting profile is then defined, equal to zero at the two extreme control points and equal to one at the central (apex) control point, with a smooth transition in between. At each station, the selected position is the weighted blend of the two boundaries: at the external control sections, the contour follows the lower edge of the wheel concentration, which lies above the absolute band bottom, where body spillover and shadows contribute occupancy but with lower persistence than the wheel; towards the curve center, the contour progressively descends to the lower contour of the full band, since there the perspective places the true contact point at the bottom of the band. The contour migrates towards the inner side of the curve, as given by the detected concavity; whether this displacement is expressed along the vertical or horizontal image axis depends on how the curve is presented in the frame and is therefore set from the camera orientation at each site. For tangent sections, where the viewing geometry does not vary along the section, the weighting term is disabled and the contour follows the lower edge of the high-concentration region uniformly along the whole section; shadows, which in this configuration lie on the upper side of the band, do not interfere with the extraction.

Two estimators of the contact point are therefore available: the lower edge of the high-concentration region and the weighted centroid of that same core. Along curves, where perspective correction applies, the lower edge is progressively blended towards the bottom of the band at the apex, as described above. The choice is governed by the height and obliquity of the bird’s-eye view perspective.

The resulting polyline is smoothed using a median filter followed by a moving average (25-point window), and isolated outliers are rejected to enforce continuity, so that the contour endpoints follow the established trajectory rather than snapping to locally distorted samples. Finally, only users whose extracted contour spans at least 70% of the section length are retained; shorter contours, corresponding to fragmented or fake tracks that do not traverse the whole section, are discarded and excluded from further analysis.

**Figure 4:**
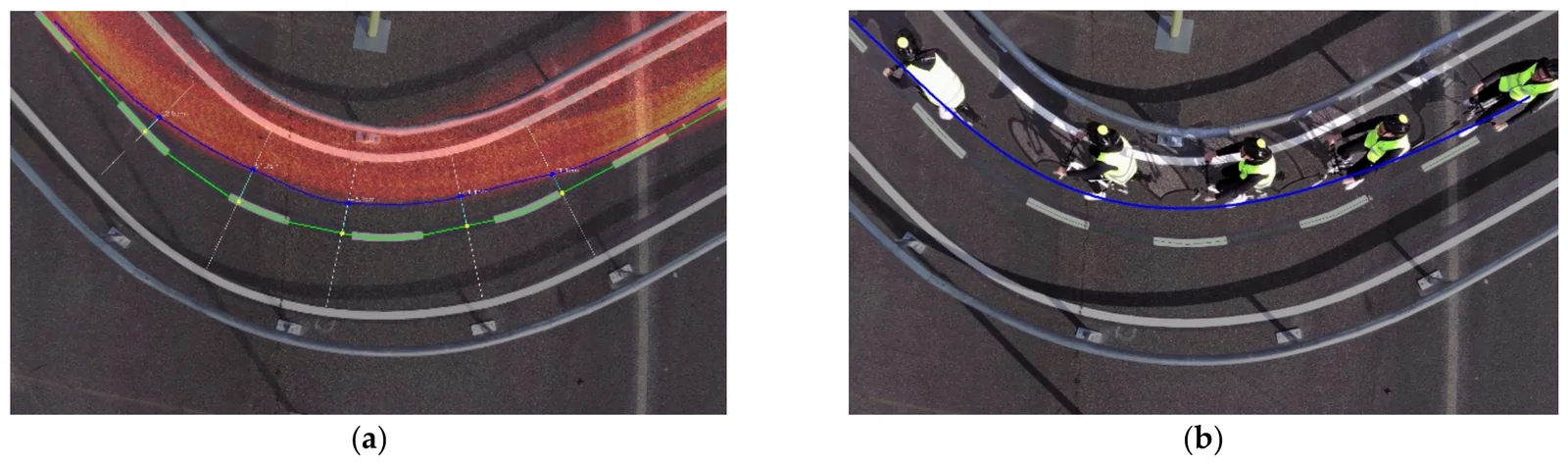
(**a**) Contour extraction and lateral position and speed quantification (**b**) Real user matching to the extracted contour.

This stage produces a continuous and smoothed estimate of the wheel-contact path of each user, stored as pixel coordinates in CSV format for the subsequent geometric analysis. Visual outputs are also generated for qualitative validation: the normalized per-user heatmap is converted into a color-coded representation and overlaid on the reference frame (Figure 4a), and the extracted contour is displayed as a polyline over both the heatmap and the reference frame (Figure 4b). All values reported in this section were established during the iterative development of the pipeline.

#### 2.1.4. Quantification of Lateral Position and Speed

Finally, the analytical stage of the pipeline consists of quantifying the lateral position and local speed of each user at the predefined control sections (e.g., PC, MP, and PT in curved sections). For each control point, the transverse measurement axis is the corrected perpendicular defined during the geometric segmentation stage, which passes through the control point and spans the section from boundary to boundary.

At each control section, the corrected perpendicular is intersected with the extracted trajectory contour of the user (Figure 4a). When an exact intersection is found, this location is assigned as the trajectory crossing point for the corresponding control section. Otherwise, a fallback sequence is applied: the contour point best aligned with the perpendicular direction is selected, provided that its deviation from the perpendicular line does not exceed 35 pixels; if no such point exists, the heatmap band is sampled directly along the perpendicular to locate the boundary of the occupied region. Control sections for which no measurement point can be established are left empty in the output files. The crossing point is subsequently linked to the closest sample of the tracked trajectory of the same user, and the frame index associated with this sample is assigned as the crossing frame, providing a consistent spatiotemporal reference for the speed computation.

The lateral position is defined as the signed distance from the control point on the centerline to the crossing point. Hereinafter, the terms lateral position and distance are used interchangeably to refer to this magnitude, with the latter used particularly when reporting measured values and their errors in the validation figures and tables. The sign convention was established according to the direction of travel: positive values indicate that the user remains within the intended lane, whereas negative values indicate encroachment into the opposite lane. Therefore, the lateral distance not only quantifies the magnitude of the deviation from the centerline, but also identifies the side of the lane in which the user is located.

The magnitude is computed as the projection of the vector connecting the control point and the crossing point onto the unit vector of the perpendicular direction. This distance is first obtained in pixels and then converted into centimeters using the pixel-to-metric scale defined during segmentation (Section 2.1.1). The sign is assigned automatically from the section geometry and the direction of travel, so that no manual convention is required for each section or riding direction. The direction of travel is derived from the net horizontal displacement of the user’s trajectory between its first and last frames. With the image origin located at the top-left corner, the sign is obtained from the combination of the net horizontal displacement and the vertical offset between the contour and the centerline. The same rule applies to tangent sections, where each direction of travel leaves its footprint on its corresponding side of the centerline.

Finally, the instantaneous speed of each user is estimated at every control section as a moving average centered on the crossing frame. A temporal window spanning ±5 frames is defined, and the cumulative path length of the trajectory points contained within this window is computed in pixels, converted into centimeters through the spatial scale, and divided by the elapsed time obtained from the video frame rate. The resulting speed is expressed in km/h.

For each valid user, the pipeline produces a single composite image combining the reference frame, the per-user heatmap, the extracted trajectory, the control geometry, and the crossing points annotated with their clearance values in centimeters, together with one CSV row per control section containing the control coordinates, the intersection coordinates, the lateral position in pixels and centimeters, and the speed in km/h. Global outputs are also stored, including the accumulated heatmap of all users, the trajectory plot, and a record of the method configuration and parameters used in the run.

### 2.2. Validation

#### 2.2.1. Validation Design and Experimental Sections

The validation procedure was designed to assess the accuracy of the pipeline in estimating the two main output variables: lateral position and speed. To this end, experimental trials were conducted on two types of sections: a tangent section and a curved section. These configurations allowed the pipeline to be evaluated under different geometric conditions, including both a controlled tangent alignment and a more complex curved trajectory.

A sample of three volunteers performed repeated riding tasks along predefined reference paths. In the tangent section, each participant completed 10 passing maneuvers. In the curved section, each participant completed 24 passing maneuvers, with 12 in each direction of circulation. Both directions were retained in the curved configuration because the direction of travel may affect the way users negotiate the curve, given the more complex trajectory imposed by the geometry.

All valid observations were retained for each validation scenario instead of subsampling the datasets to a common size. As the analyses were conducted independently for each variable and section type, and no direct inferential comparison between sections was intended, the different sample sizes did not affect the validity of the within-section validation results.

**Figure 5:**
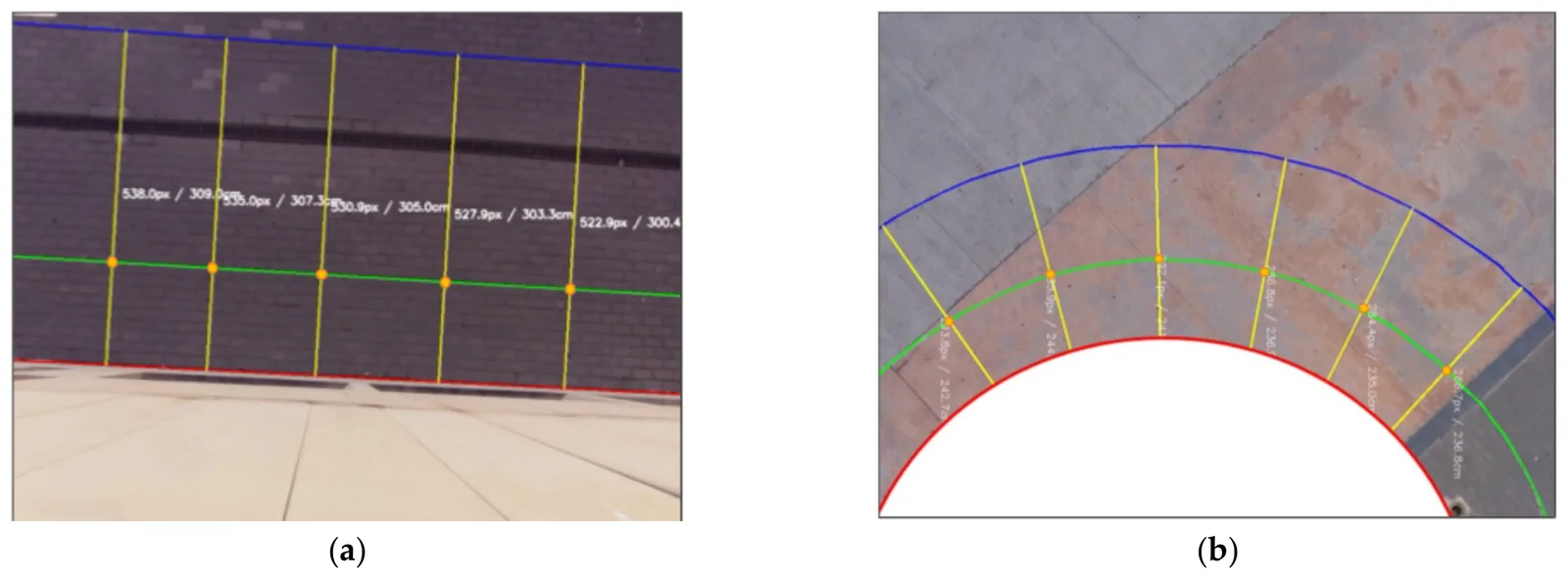
Experimental sections for validation. (**a**) Tangent section; (**b**) Curved section.

The trials were recorded from a bird’s-eye-view perspective at 30 FPS, with a video resolution of 1920 × 1080 pixels. Videos were recorded at independent outdoor locations, on two different days and at different times between 10:00 am and 15:00. The video duration was 15 min and 50 s for the tangent section and 30 min and 38 s for the curved section. The camera height was 5 m for the tangent section and 8 m for the curved section. The resulting pixel-to-metric calibration yielded processing scales of 1.74 px/cm in the tangent section and 1.39 px/cm in the curved section. In both sections, control points were defined along the reference path, as shown in Figure 5a,b. These points correspond to the locations where lateral position and speed were extracted by the pipeline, following the procedure described in Section 2.1.

The tangent section was processed with the lower edge of the high-concentration region, and the curved section was processed with the weighted centroid, as described in Section 2.1.3, based on the field of view obtained for each site and derived from the camera height for each section.

Results are presented by variable rather than by section, as the main objective of the validation was to assess the ability of the pipeline to estimate lateral position and speed. Within each variable, results are reported separately for the tangent and curved sections to evaluate the influence of section geometry on measurement performance.

#### 2.2.2. Reference Measurements

##### Lateral Position Reference Measurements

The reference measurements were defined according to the characteristics of each validation section. In the tangent section, the pipeline-derived contour was superimposed on the cobblestone mesh, which provided a spatial reference with known dimensions. The cobblestone units used as reference elements had dimensions of 20 cm in width and 10 cm in height. This allowed the distances estimated for lateral position by the pipeline to be directly compared with the corresponding real distances measured on the reference mesh.

In the curved section, the validation was based on a fixed known reference distance of 140 cm. Only observations in which the riding task was properly performed, with the user circulating along the predefined reference line, were retained for the analysis. The pipeline-derived distance at each control point was then compared with the fixed reference distance.

For both sections, the distance error was defined as the difference between the pipeline-derived distance and the corresponding ground truth measurement:

(1) ∆ Distance = Pipeline distance measurements−Ground truth distance measurements

This difference was used as the main variable for assessing distance measurement accuracy.

##### Speed Reference Measurements

For speed validation, the reference measurement was obtained from the e-scooter in both experimental sections. Participants were instructed to perform the riding tasks at a constant speed, which was facilitated by the e-scooter cruise-control function.

Before using the e-scooter readings as the reference for the validation, an independent verification of its speed measurement was conducted. The device was a Xiaomi Electric Scooter 4 Pro (first generation) (Xiaomi Communications Co., Ltd., Beijing, China), with a maximum speed of 25 km/h, a maximum power output of 700 W and a wheel width of approximately 2 in. (5.08 cm). The same unit was subsequently used in the validation trials. One volunteer performed 30 passing maneuvers over a fixed travel distance of 100 m at three stabilized cruising speed levels (10, 15, and 20 km/h), with 10 repetitions per level, recording the travel time of each maneuver. A conventional reference speed was then computed as:

(2) $$V=\frac{l}{t}$$

where *l* is the length of the trial in meters (100 m) and *t* is the travel time in seconds. The resulting speed was converted to km/h and compared with the speed displayed by the e-scooter.

(3) ∆V=Vlt−Ve-scooter

where Vlt is the speed calculated from the distance–time relationship, and $V_{scooter}$ is the speed displayed by the e-scooter. Therefore, negative values indicate that the e-scooter reading was higher than the distance–time estimate.

The verification showed a low overall bias with limited dispersion across the three speed levels (Table 1). The largest deviations appeared at the highest speed level, where the e-scooter tended to report slightly higher speeds than the distance–time estimates, although the differences remained low in practical terms.

The tolerance-based assessment (Table 2) further supported the use of the e-scooter as a practical reference, with nearly all readings within ±1.0 km/h of the distance–time estimates. On this basis, the e-scooter speed readings were considered a sufficiently consistent reference for validating the pipeline-derived speeds.

For the subsequent validation, users were assumed to circulate at an approximately constant speed during each maneuver. Therefore, the mean of the pipeline-derived speeds obtained at the five control points (Figure 2b) was computed for each pass, allowing direct comparison with the corresponding e-scooter speed measurement.

The speed error was defined as the difference between the mean pipeline-derived speed and the e-scooter measurement:

(4) ∆ Speed=Mean pipeline speed measurements−e-scooter speed measurements

This difference was used as the main variable for assessing speed measurement accuracy.

#### 2.2.3. Error Metrics and Tolerance-Based Assessment

For each validation variable, descriptive statistics were first computed for the paired measurements before calculating the errors. This preliminary analysis was performed separately for the pipeline-derived measurements and the corresponding reference measurements, in order to characterize the distribution of each sample. The mean, median, standard deviation, minimum, and maximum values were obtained for both lateral position and speed measurements.

Subsequently, the error distributions were analyzed using the paired differences defined in the previous sections. For both variables, the mean difference was used to quantify the average bias between the pipeline and the reference measurement. The standard deviation and median of the differences were also computed to describe the dispersion and central tendency of the error distribution.

In addition, two complementary error metrics were calculated: the mean absolute error (MAE) and the root mean square error (RMSE). MAE was used to quantify the average magnitude of the error regardless of its sign, while RMSE provided a measure more sensitive to larger deviations. These metrics allowed the magnitude and variability of the pipeline error to be assessed for both lateral position and speed.

Finally, a tolerance-based assessment was performed to evaluate the practical relevance of the observed errors. Since lateral position and speed have different measurement characteristics and precision requirements, different tolerance thresholds were defined for each variable. For speed, tolerance thresholds of ±0.5, ±1.0, ±2.0, and ±3.6 km/h were considered, with ±2.0 km/h used as the main practical threshold and ±3.6 km/h corresponding to ±1 m/s. This limit was established because the e-scooter’s speedometer presents a maximum deviation of 1.405 km/h for a speed of 20 km/h, as presented in Table 1. Therefore, tolerance levels below this value would reflect the uncertainty of the reference rather than the accuracy of the tool. For lateral position, tolerance thresholds of ±2, ±5, and ±10 cm were considered, with ±5 cm used as the main practical threshold. Similarly, this limit was defined based on the physical width of the e-scooter wheel, which is 5 cm, as described in the Section Speed Reference Measurements, and is representative of micromobility vehicles, including urban bicycles. Therefore, the wheel–pavement contact area spans approximately this distance, and any point within this range can be considered representative of the lateral position being measured.

Overall, these thresholds were selected to represent increasing levels of acceptable deviation, accounting for both the physical scale of the measurement and the expected accuracy of the reference and tracking procedure.

#### 2.2.4. Statistical Analysis

Inferential statistical analysis was performed to determine whether the observed paired differences between the pipeline-derived measurements and the corresponding reference measurements were statistically different from zero. This analysis was used to identify the presence of systematic bias in the pipeline estimates for both lateral position and speed.

Prior to hypothesis testing, the normality of the paired differences was assessed. The Shapiro–Wilk test [27] was used for samples with fewer than 50 observations, whereas the Kolmogorov–Smirnov test [28] was applied for samples with 50 or more observations. When normality was not rejected, a two-sided paired *t*-test was performed to evaluate whether the mean paired difference differed significantly from zero. The significance level was set at alpha = 0.05.

In addition to inferential testing, graphical agreement analyses were used according to the characteristics of the reference measurements. Bland–Altman analysis was applied when both methods produced variable measurements across observations. This analysis was used to quantify the mean bias and the 95% limits of agreement, providing information on the practical agreement between methods and their potential interchangeability. When the reference value was fixed, graphical assessment focused instead on the distribution of the measurement errors, since this approach more directly represented the deviations of the pipeline estimates from the known reference value.

## 3. Results

### 3.1. Lateral Position

#### 3.1.1. Tangent Section Lateral Position Validation 

Pipeline-derived lateral position measurements were compared with the corresponding ground truth distances obtained from the reference mesh. A total of 140 valid paired observations were analyzed. As shown in Table 3, both methods presented a very similar central tendency, with mean values differing by less than 1 cm, and comparable dispersion, which was slightly lower in the pipeline sample.

**Figure 6:**
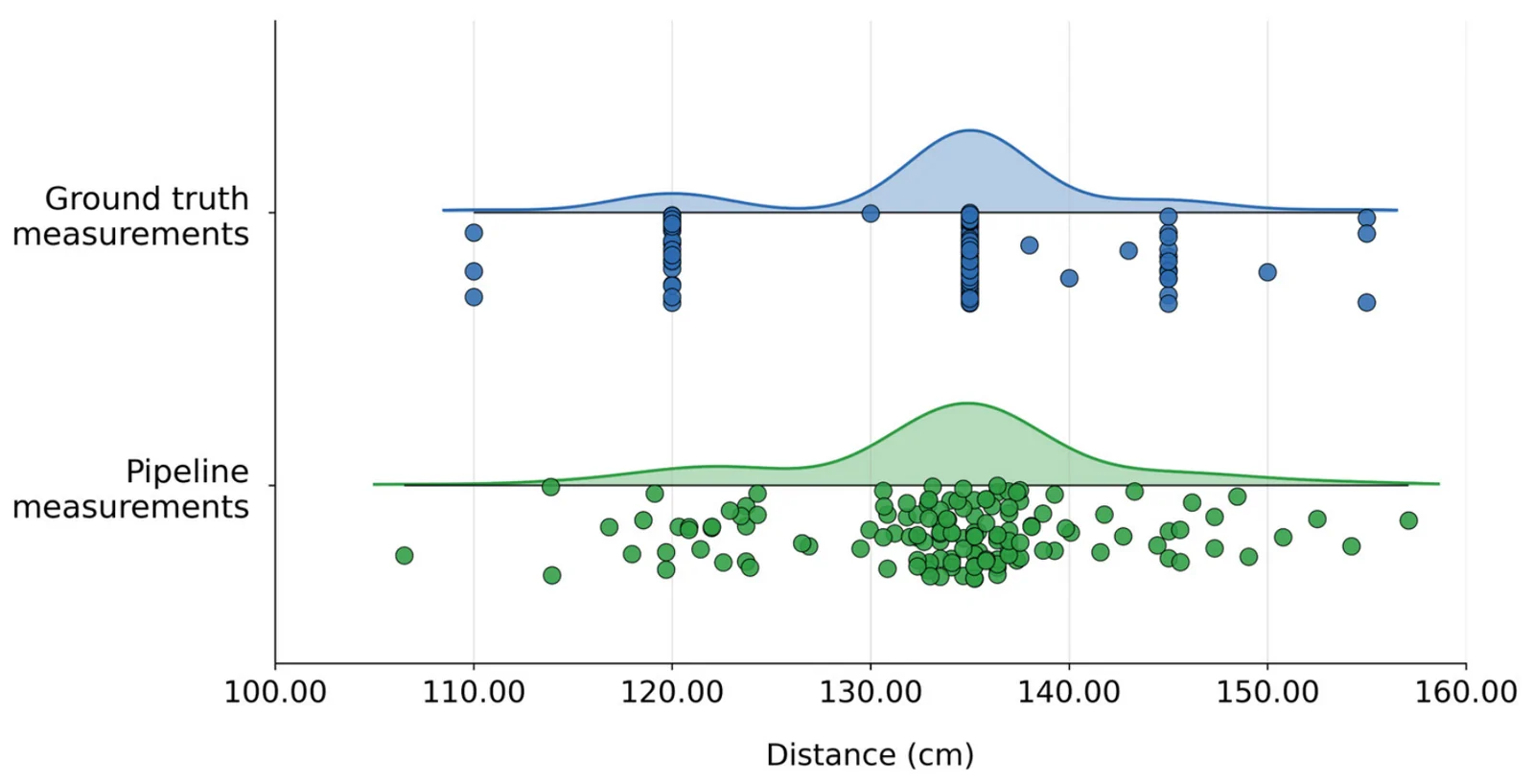
Distribution of distance measurements in the tangent section.

The distributions of the ground truth and pipeline-derived measurements are shown in Figure 6. Both methods showed a similar central pattern, with most observations concentrated around 130–140 cm; the pipeline distribution was slightly more compact and marginally shifted towards higher values, consistent with the small mean overestimation reported in Table 3.

**Figure 7:**
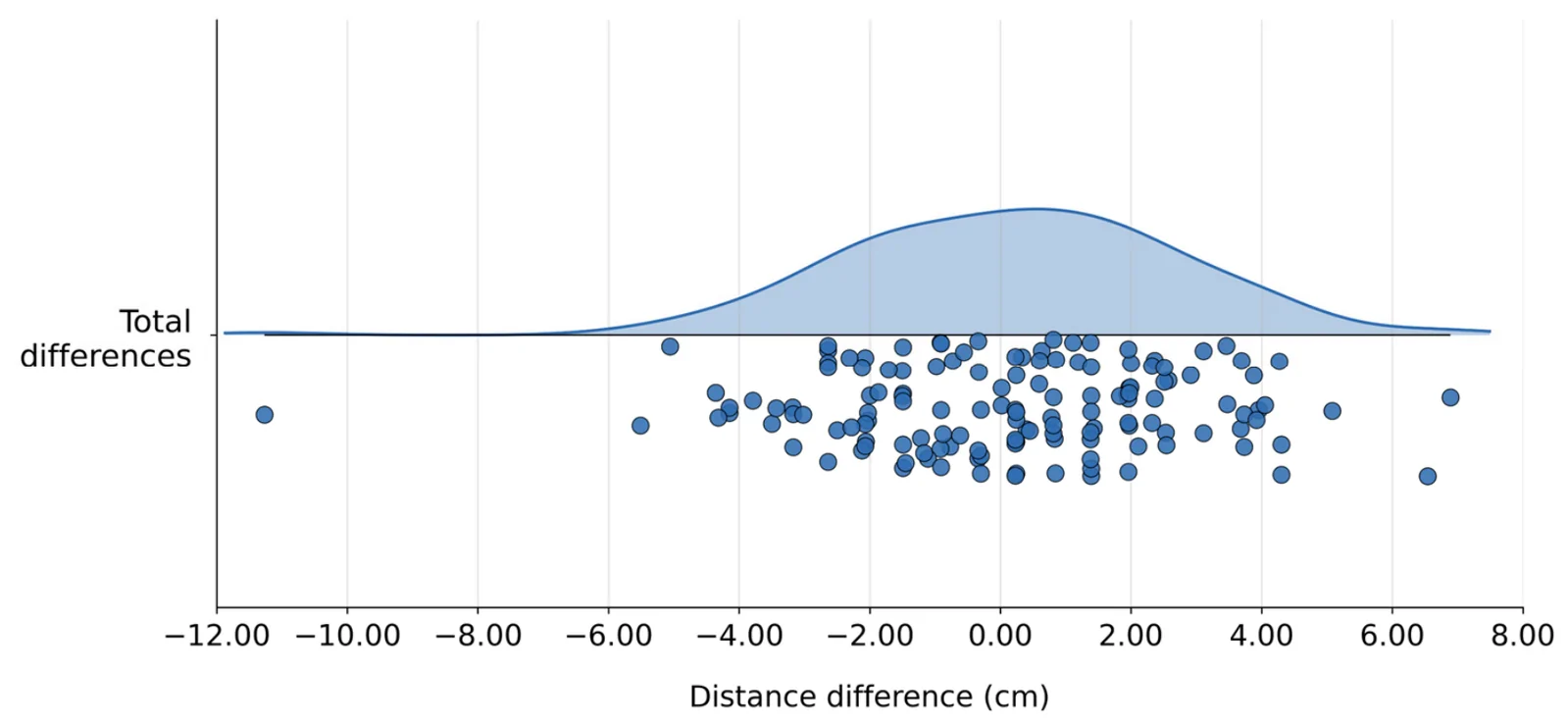
Distribution of distance errors in the tangent section.

In addition, the distribution of the distance errors is shown in Figure 7. Most differences were concentrated around zero, with a slight positive shift and a dispersion of about 2 cm, and even the extreme deviations remained within a narrow interval around zero (Table 3).

The MAE and RMSE were 2.022 cm and 2.577 cm, respectively, confirming the limited magnitude of the distance errors. Regarding tolerance thresholds, 57.9% of the observations were within ±2 cm, 95.7% were within ±5 cm, and 99.3% were within ±10 cm. These results show that, although a slight positive bias was observed, almost all measurements remained within the main practical tolerance threshold of ±5 cm.

Normality of the paired differences was assessed using the Kolmogorov–Smirnov test. The test did not reject the null hypothesis of normality (*p* = 0.756). Therefore, a two-sided paired *t*-test was applied. The mean difference was not statistically different from zero (t (139) = 0.673, *p* = 0.502), with a 95% confidence interval ranging from −0.285 to 0.578 cm. This result indicates that there was no statistically significant evidence of systematic difference between the distance estimated by the pipeline and the ground truth reference.

**Figure 8:**
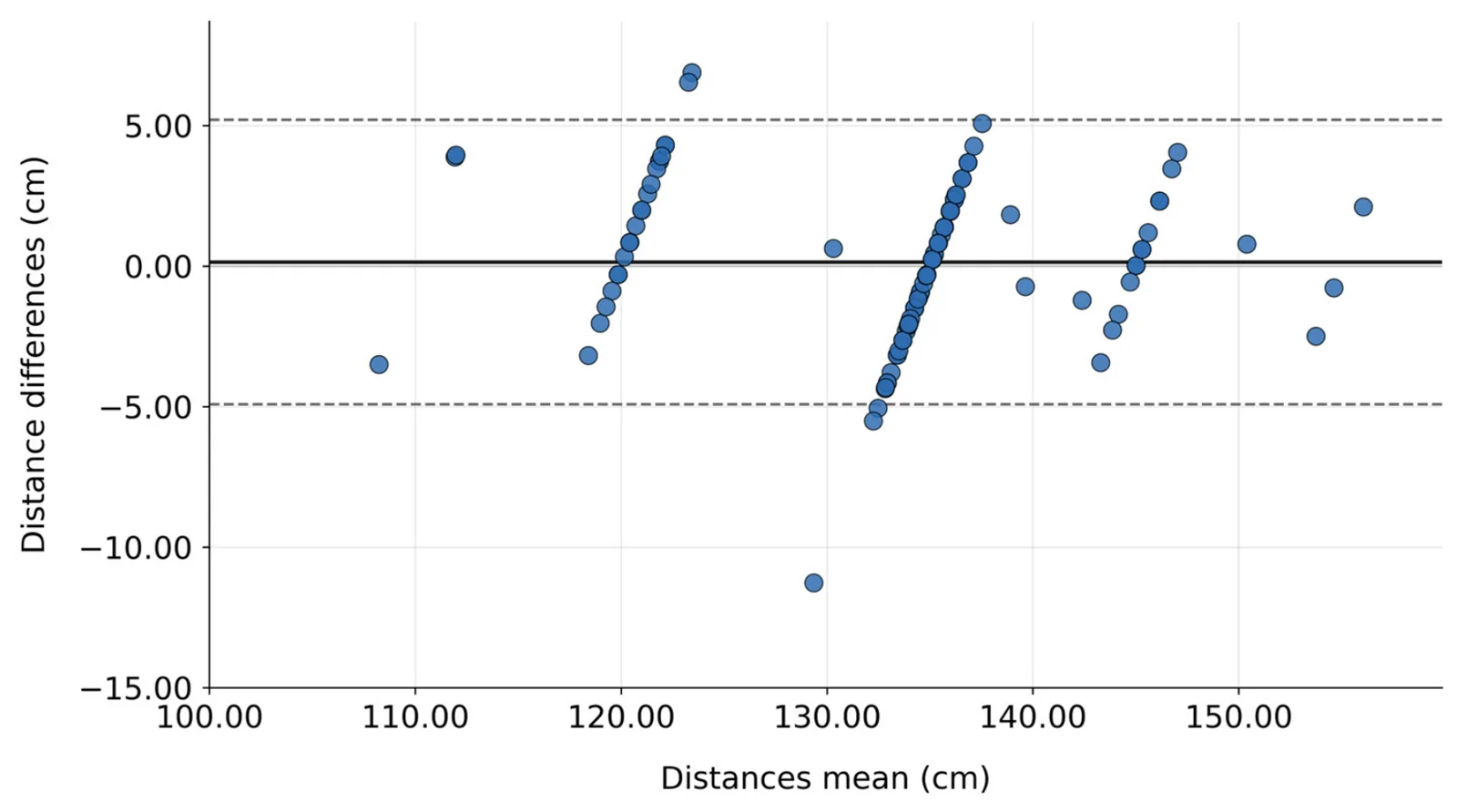
Bland–Altman plot for distance in the tangent section.

The Bland–Altman analysis, presented in Figure 8, showed a mean bias of 0.25 cm, with 95% limits of agreement from −4.98 cm to 5.49 cm. Of the one hundred and forty valid observations, three were located outside the limits of agreement, meaning that 97.8% of the differences remained within the expected agreement interval. No clear visual evidence of proportional bias was observed, as the differences did not systematically increase or decrease with the magnitude of the measured distance. Overall, the pipeline showed consistency with the ground truth measurements for lateral distance estimation in the tangent section.

#### 3.1.2. Curved Section Lateral Position Validation

For the curved section, pipeline-derived lateral position measurements were compared with the fixed ground truth distance of 140 cm. A total of 295 valid observations were analyzed. As shown in Table 4, the sample was centered below the reference value and presented a wider dispersion than in the tangent section, with measured values spanning a range of nearly 30 cm.

**Figure 9:**
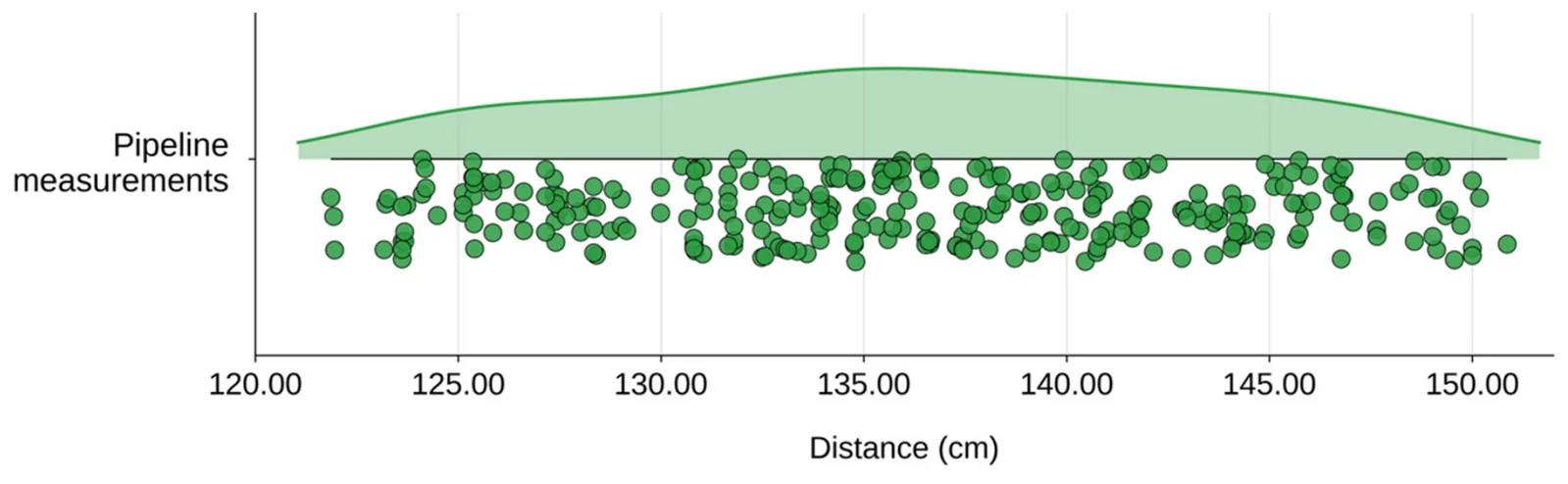
Distribution of distance measurements in the curve section.

**Figure 10:**
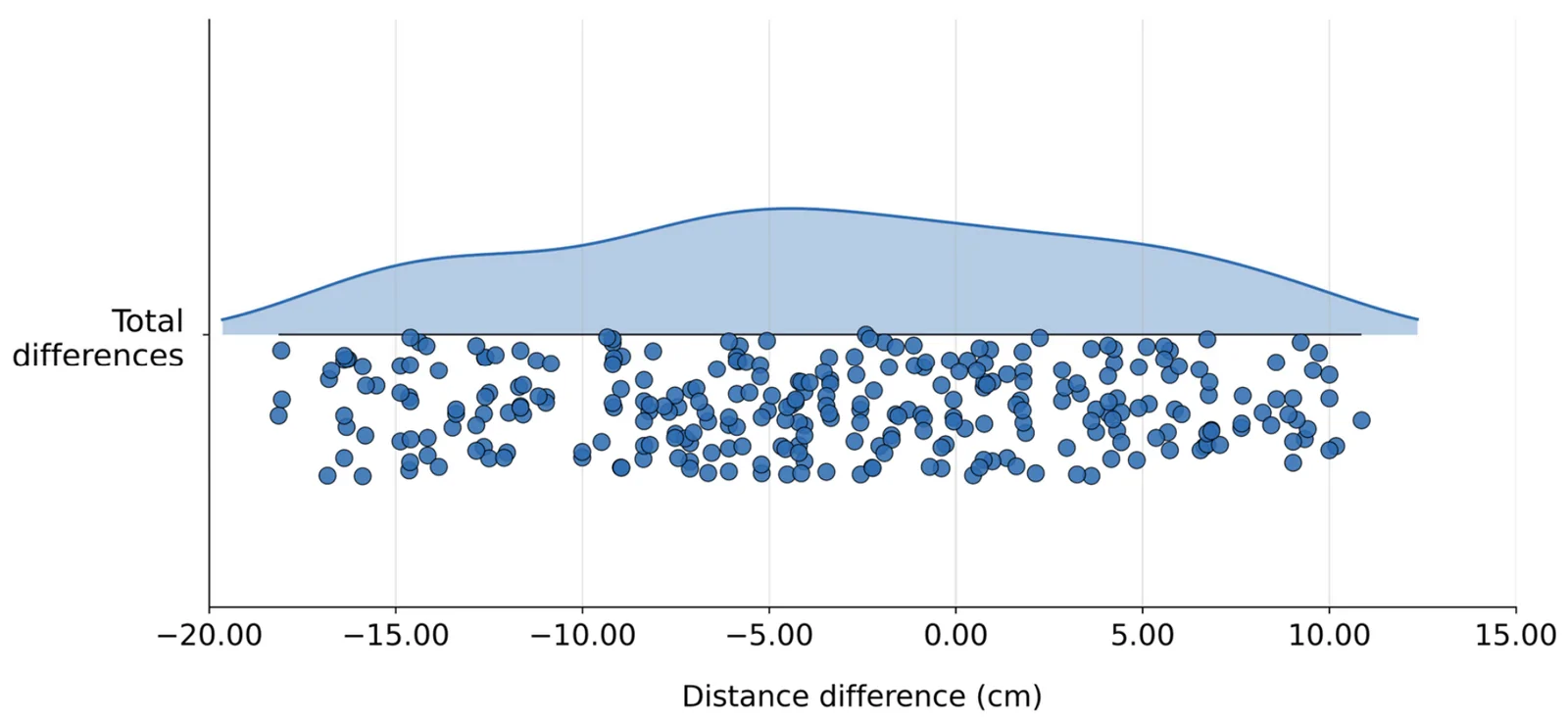
Distribution of distance errors in the curve section.

The distribution of the pipeline-derived distance measurements is shown in Figure 9, while the distribution of the distance errors is shown in Figure 10. Since the ground truth was fixed at 140 cm, the error distribution provides a direct representation of the deviations from the known reference value. Most differences were shifted towards negative values, indicating that the pipeline tended to underestimate the reference distance in the curved section.

The mean and median differences were negative and practically identical, confirming a systematic underestimation of the fixed reference distance, with a dispersion roughly three times larger than in the tangent section (Table 4). In terms of absolute error, the MAE was 6.77 cm and the RMSE was 8.20 cm, showing that although the average bias was moderate, some observations presented larger deviations from the reference.

Regarding tolerance thresholds, 17.6% of the observations were within ±2 cm, 41.7% were within ±5 cm, and 77.6% were within ±10 cm. These results indicate that the curved configuration produced higher distance errors than the tangent section, particularly under the stricter tolerance thresholds.

Normality of the paired differences was assessed using the Kolmogorov–Smirnov test. The test did not reject the null hypothesis of normality (*p* = 0.321). Therefore, a two-sided paired *t*-test was applied. The mean difference was statistically different from zero (t (294) = −8.14, *p* < 0.001), with a 95% confidence interval from −4.36 to −2.66 cm. This result confirms the presence of a statistically significant negative bias in the curved section.

Unlike the tangent section, Bland–Altman analysis was not applied in this case because the reference distance was fixed. Instead, the graphical assessment focused on the error distribution, which directly represents the deviations of the pipeline-derived measurements from the known reference distance. Overall, the pipeline systematically underestimated the distance in the curved section, with greater dispersion than in the straight configuration.

#### 3.1.3. Aggregated Analysis

An aggregate analysis of the distance measurements was performed for both tangent and curve sections. As shown in Figure 5a, for the tangent section, each passing maneuver covers the five individual control points. Consequently, 28 passing maneuvers were analyzed. The mean distance was calculated for each maneuver, and the resulting descriptive statistics are presented in Table 5.

As a result, the mean error was similar to that obtained from the individual observation analysis. The dispersion was lower; however, this reduction is partly due to averaging positive and negative signed errors. In addition, the MAE and RMSE were 1.113 cm and 1.363 cm, respectively, indicating again a limited magnitude of the distance errors.

For the curve section, six control points were established, as shown in Figure 5b. Passing maneuvers with at least four valid control-point observations were included in the analysis, resulting in a sample of 50 passing maneuvers. The corresponding descriptive statistics are presented in Table 6. As a result, an error of 3.76 cm remained when comparing the passing measurements with the observations, as also reflected by the MAE and RMSE values of 5.184 cm and 6.034 cm, respectively, which indicate a relatively limited error magnitude, although higher than that observed for the tangent section.

Overall, the aggregated analysis preserved a similar error bias in both sections, while reducing the dispersion due to the averaging of positive and negative errors within each passing maneuver.

### 3.2. Speed

#### 3.2.1. Tangent Section Speed Validation 

**Figure 11:**
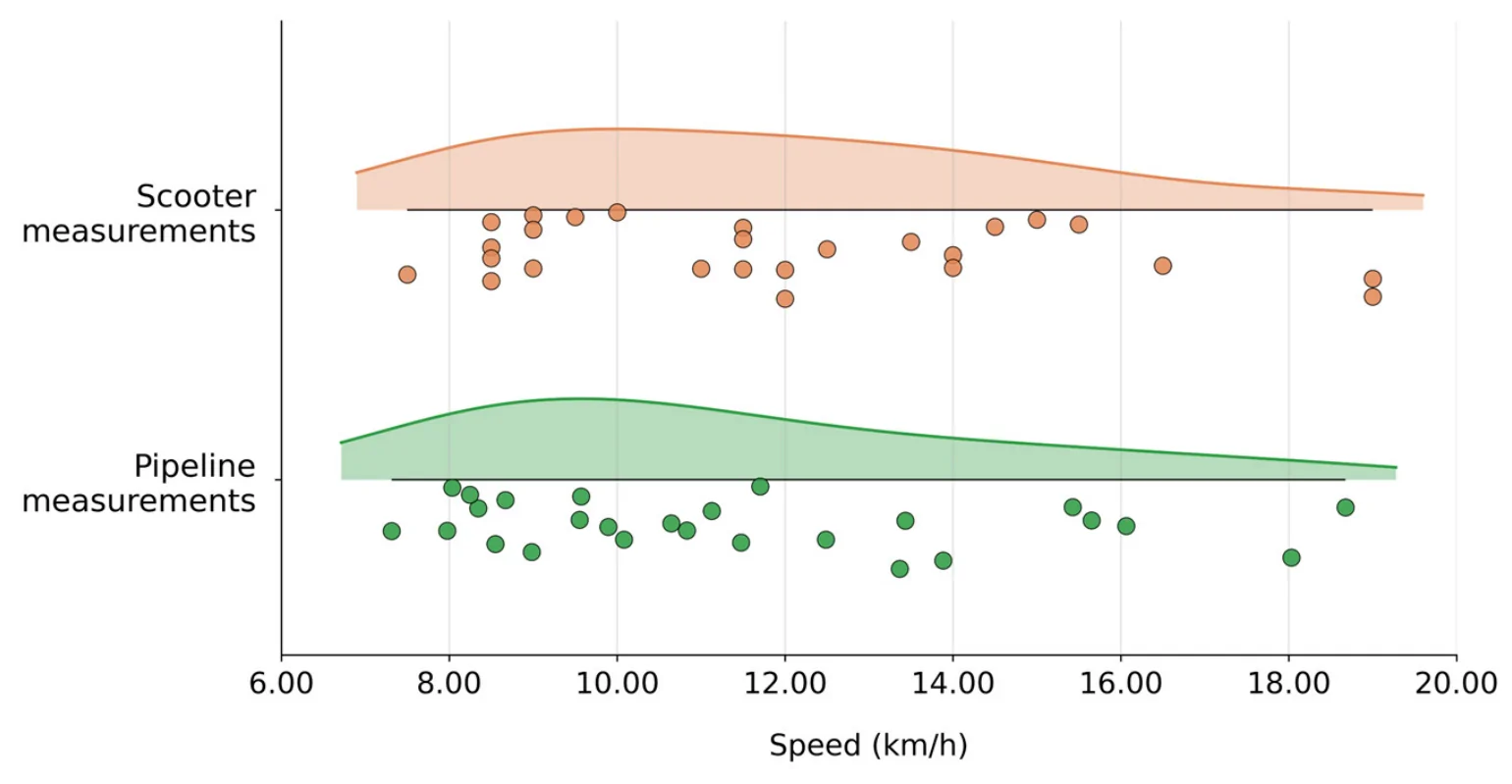
Distribution of speed measurements in the tangent section.

For the tangent section, speed validation was performed by comparing the e-scooter speed measurements with the mean speed estimated by the pipeline for each maneuver. A total of 26 valid paired observations were analyzed. Both approaches presented almost identical dispersion and a difference between mean speeds of about half a km/h, with the pipeline being slightly higher (Table 7). The distributions of both speed measurements are shown in Figure 11, indicating a similar overall range and central tendency between the two measurement approaches.

**Figure 12:**
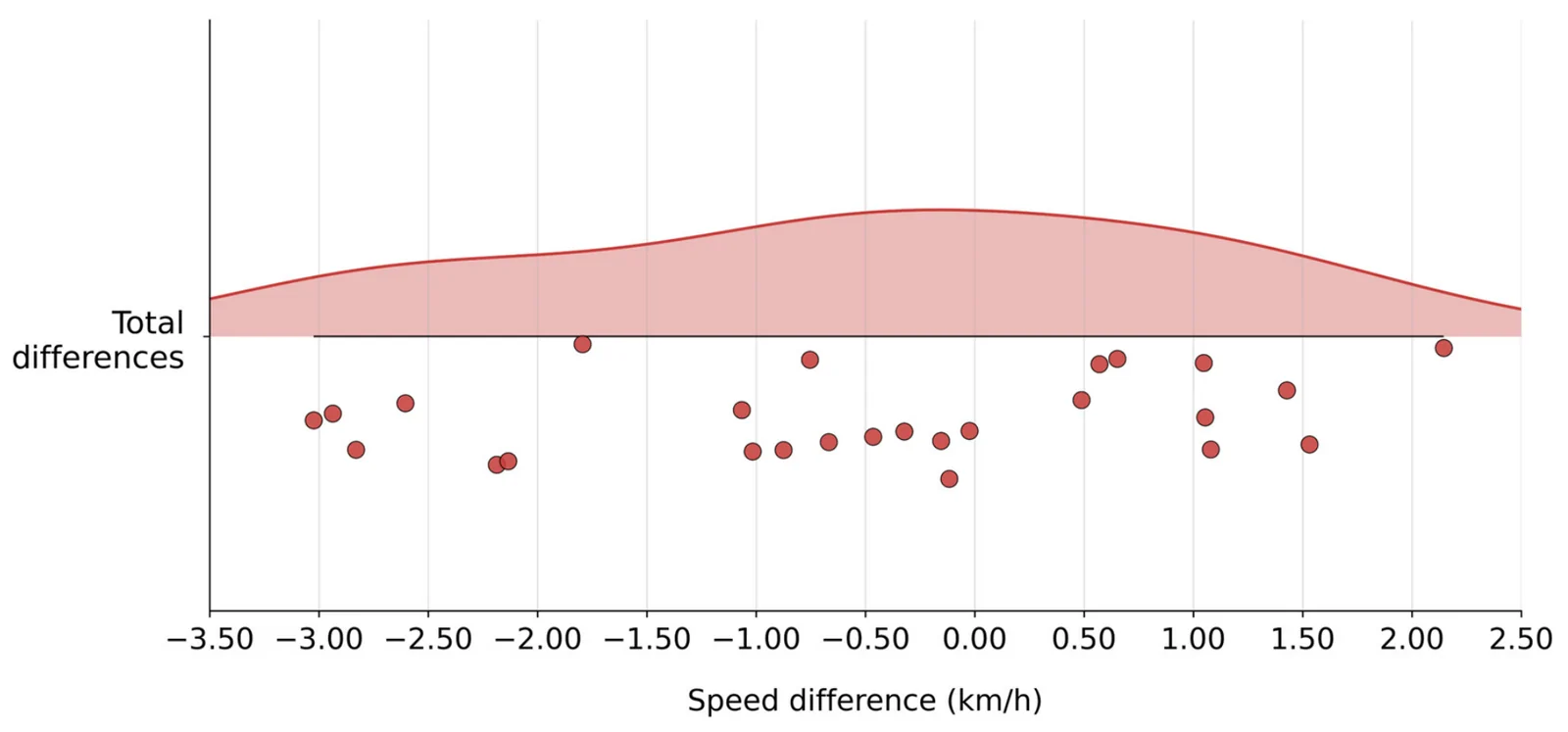
Distribution of speed errors in the tangent section.

The distribution of the speed errors is shown in Figure 12. The differences were slightly shifted towards positive values, indicating that the pipeline tended to estimate somewhat higher speeds than the e-scooter, with a moderate dispersion of the errors (Table 5). In terms of absolute error, the MAE was 1.26 km/h and the RMSE was 1.55 km/h.

Regarding tolerance thresholds, 42.3% of the observations were within ±1.0 km/h, 73.1% were within ±2.0 km/h, and all observations were within ±3.6 km/h. These results indicate that the consistency between the pipeline and the e-scooter improved as the tolerance threshold increased. Although less than half of the observations were within the strict ±1.0 km/h threshold, most observations were contained within ±2.0 km/h, and all measurements remained within the broader ±3.6 km/h criterion.

Normality of the paired differences was assessed using the Shapiro–Wilk test. The test did not reject the null hypothesis of normality (*p* = 0.355). Therefore, a two-sided paired *t*-test was applied. The mean difference was not statistically different from zero at the 95% confidence level (t (25) = 1.695, *p* = 0.103), with a 95% confidence interval ranging from −1.105 to 0.107 km/h. This indicates that there was no statistically significant evidence of systematic difference between the average speed estimated by the pipeline and the speed recorded by the e-scooter in the tangent section.

**Figure 13:**
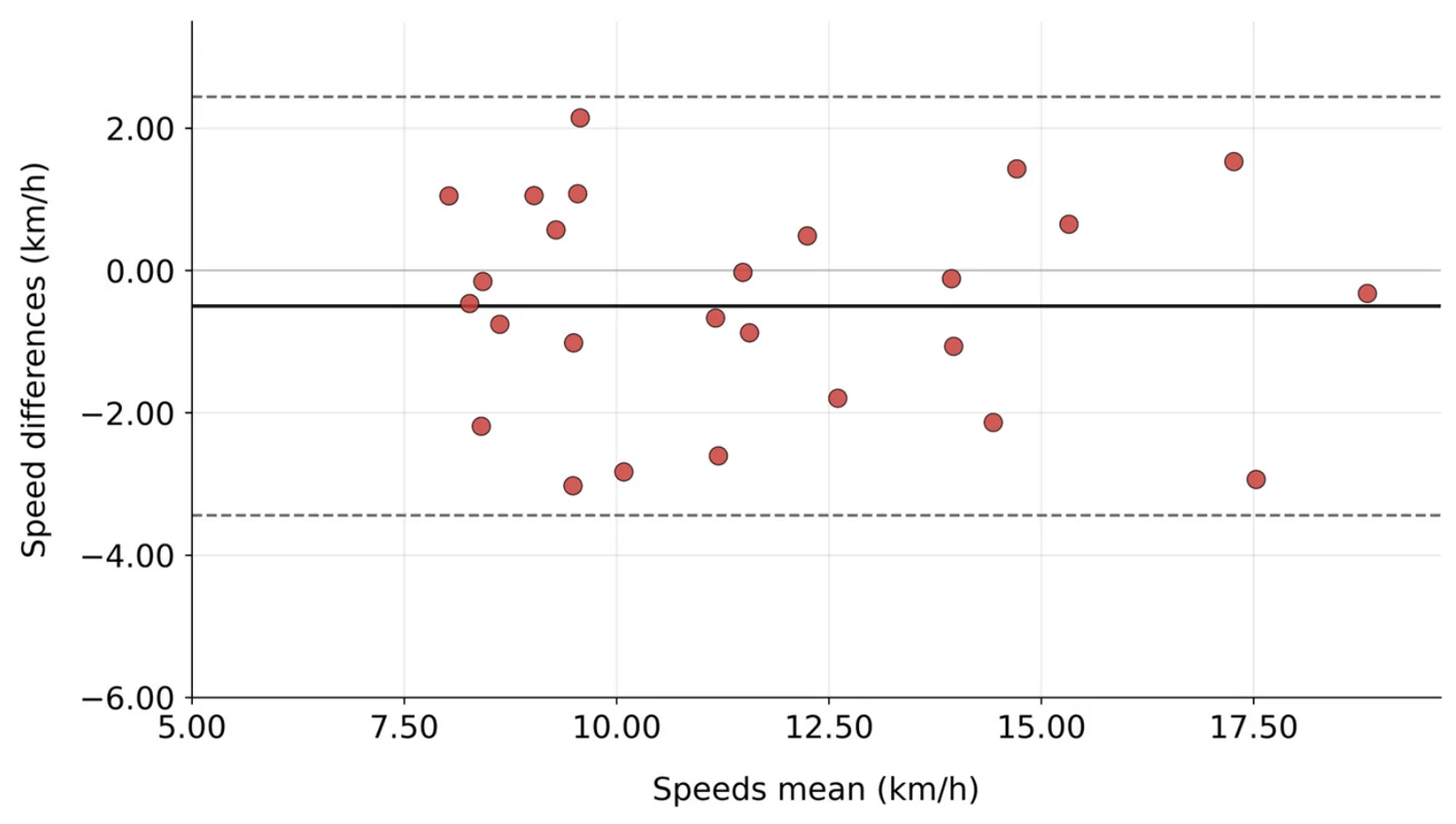
Bland–Altman plot for speed in the tangent section.

The Bland–Altman analysis showed a mean bias of −0.49 km/h, with 95% limits of agreement from −3.44 km/h to 2.44 km/h (Figure 13). No clear visual pattern was observed in the Bland–Altman plot, suggesting no evident proportional bias. The limits of agreement were contained within the broader tolerance of ±3.6 km/h but exceeded the main practical threshold of ±2.0 km/h. Therefore, the consistency between both approaches can be considered reasonable for average speed estimation. 

#### 3.2.2. Curved Section Speed Validation

**Figure 14:**
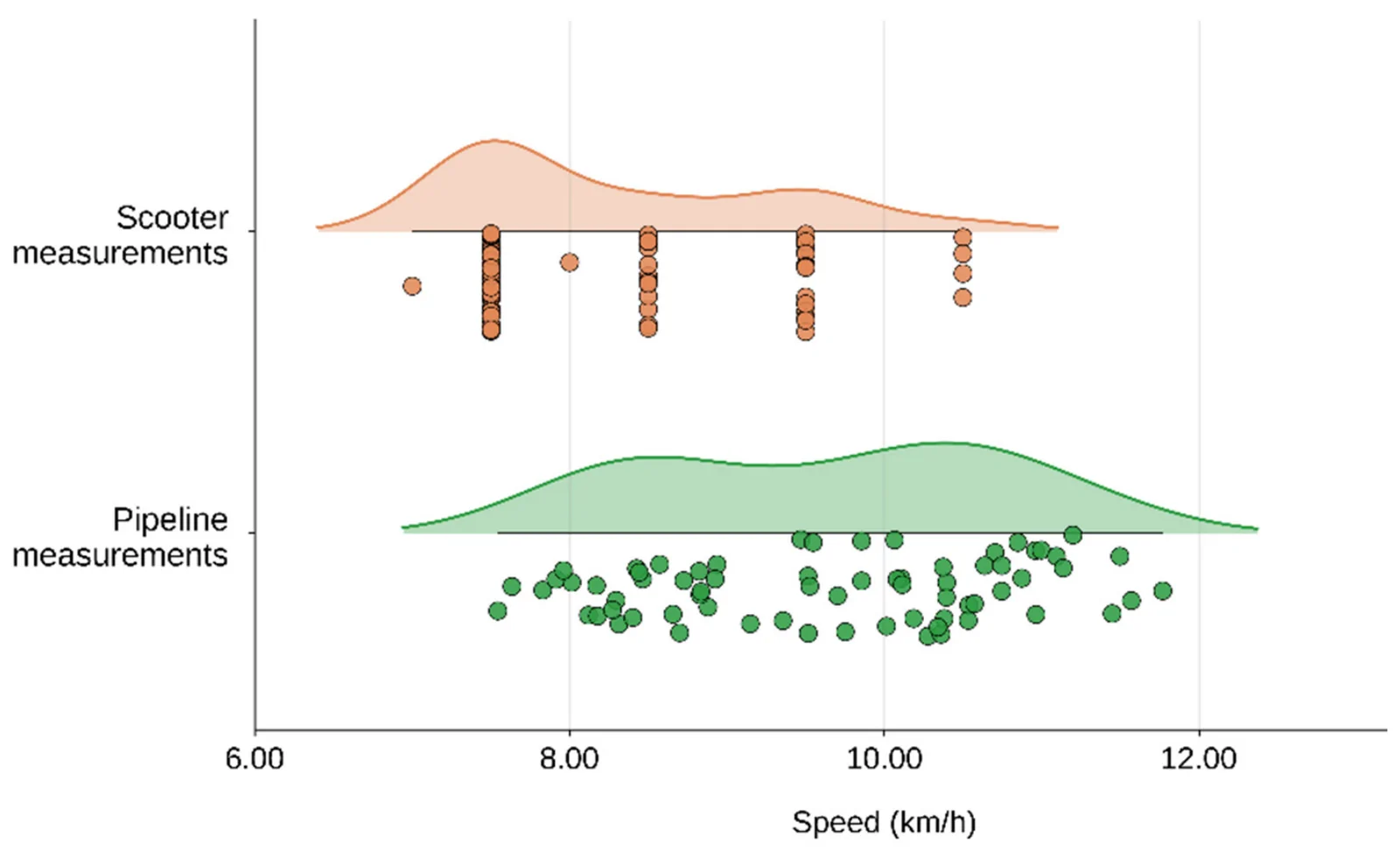
Distribution of speed measurements in the curve section.

For the curved section, speed validation was performed following the same procedure as in the tangent section, comparing the e-scooter measurements with the mean speed estimated by the pipeline for each maneuver. The distributions of both speed measurements are shown in Figure 14; the pipeline distribution was shifted towards higher values, indicating that the pipeline generally estimated higher speeds in this section.

**Figure 15:**
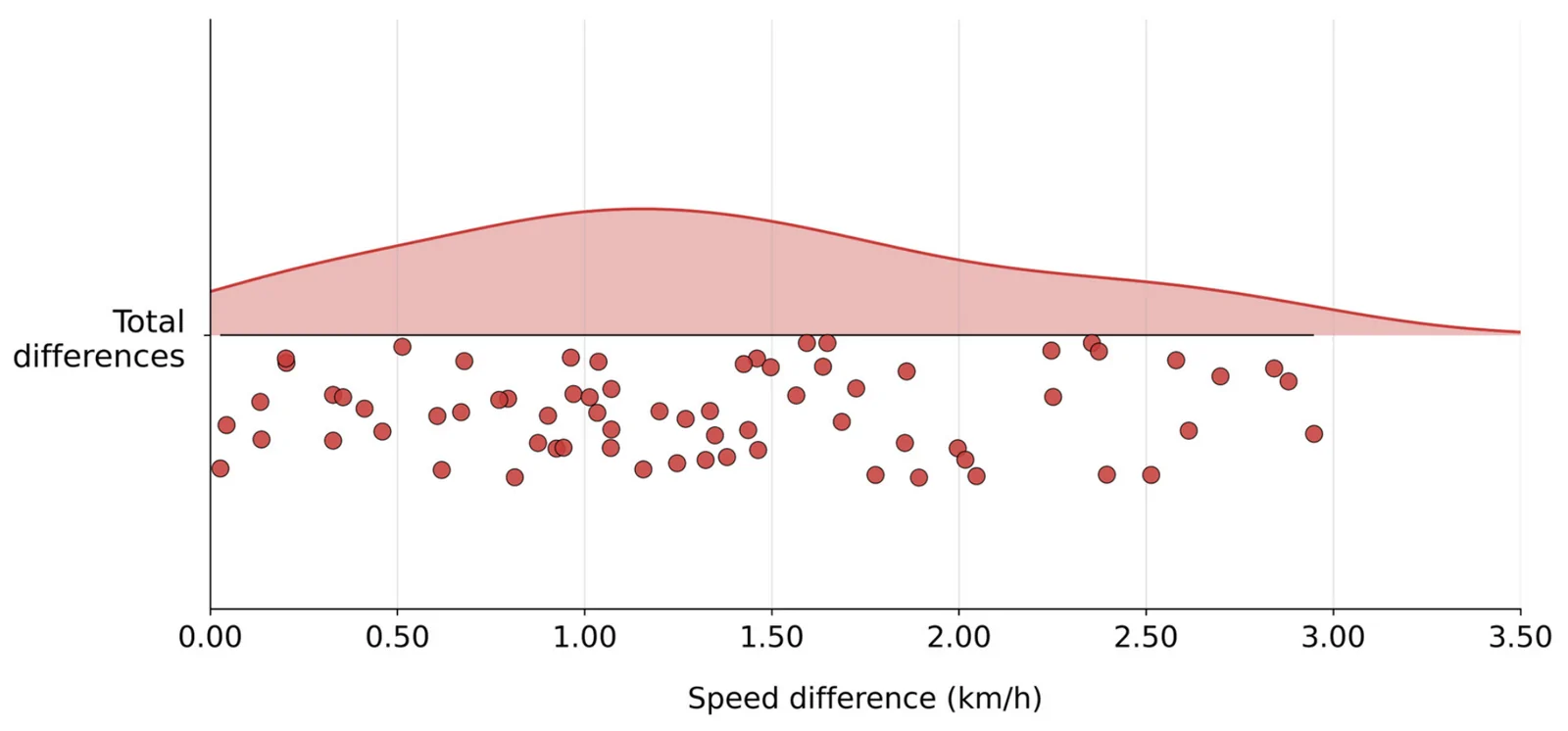
Distribution of speed errors in the curve section.

A total of 69 valid paired observations were analyzed. The difference between mean speeds exceeded 1 km/h, with slightly higher dispersion in the pipeline estimates than in the e-scooter readings (Table 8). The distribution of the speed errors is shown in Figure 15. All differences were positive, indicating that the pipeline consistently overestimated the e-scooter speed measurements in this section, with a limited dispersion of the errors. In terms of absolute error, the MAE was 1.326 km/h and the RMSE was 1.53 km/h.

Regarding tolerance thresholds, the agreement between the pipeline and the e-scooter was moderate under stricter tolerances and stronger under broader thresholds. A total of 15.9% of the observations were within ±0.5 km/h, 36.2% were within ±1.0 km/h, 79.7% were within ±2.0 km/h, and all observations were within ±3.6 km/h. These results show that, although the pipeline systematically overestimated speed in the curved section, the magnitude of the error remained within the broadest practical tolerance threshold for all observations.

Normality of the paired differences was assessed using the Kolmogorov–Smirnov test. The test did not reject the null hypothesis of normality (*p* = 0.752). Therefore, a two-sided paired *t*-test was applied. The mean difference was statistically different from zero (t (68) = 14.332, *p* < 0.001), with a 95% confidence interval from 1.142 to 1.511 km/h. This result confirms the presence of a statistically significant positive bias in the pipeline-derived speed estimates for the curved section.

**Figure 16:**
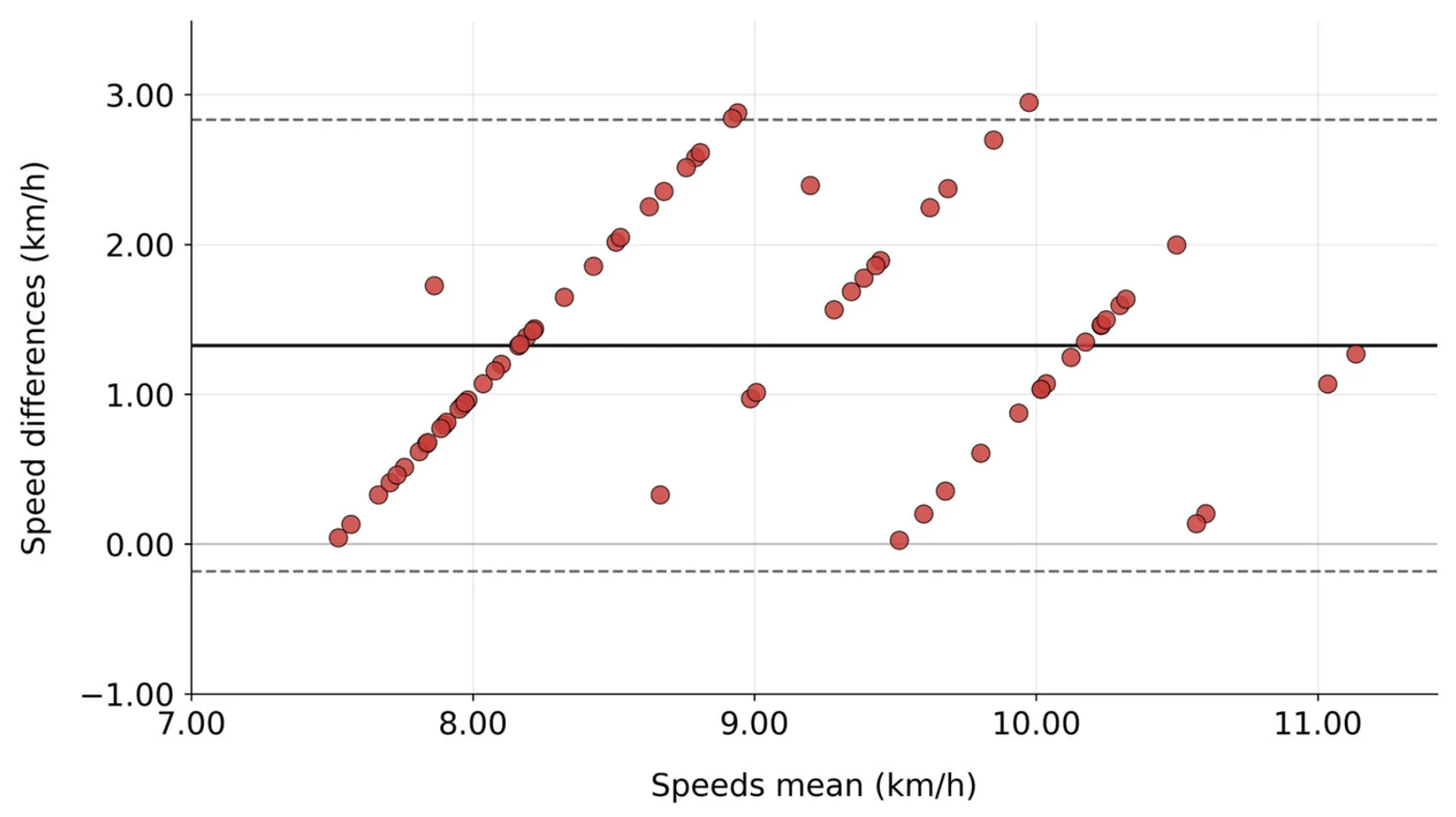
Bland–Altman plot for speed in the curve section.

The Bland–Altman analysis (Figure 16) showed that most observations were contained within the 95% limits of agreement. Together with the tolerance-based assessment, where 79.7% of the observations were within ±2.0 km/h and 100% within ±3.6 km/h, this indicates an acceptable practical concordance between the pipeline mean measurements and e-scooter measurements.

## 4. Discussion

### 4.1. Summary of Validation Performance

The validation performance of the tool for both variables and section types is summarized in Table 9. The tool reproduced the reference measurements in the tangent section, with a negligible bias for lateral position and no statistically significant difference for speed. In the curved section, systematic deviations were detected, consisting of an underestimation of the lateral position and an overestimation of the speed; part of these deviations is attributable to the greater complexity of the curved validation task, in which users had to follow a predefined reference line, rather than to the measurement procedure alone. Statistical significance was not adopted as the decisive criterion of the validation, since several biases, although statistically detectable, were small against the predefined tolerances; the practical assessment therefore relied on the magnitude of the errors and on the tolerance-based analysis, as discussed below.

### 4.2. Interpretation of Validation Results

The performance of the pipeline depended on both the analyzed variable and the geometry of the section. The tangent section presented the most favorable results. For lateral position, the pipeline reproduced the ground truth with low mean error and limited dispersion, and almost all observations remained within the ±5 cm tolerance. At the aggregate level, the same trend was observed when considering each passing maneuver.

For speed, the mean difference with respect to the e-scooter measurements was small and not statistically significant, with more than 70% of the observations within ±2.0 km/h. The Bland–Altman assessment confirms no evident proportional bias and supports the usage of the pipeline as a reasonable method for average speed estimation.

Deviations were larger in the curved section. The pipeline systematically underestimated the reference distance, with higher dispersion, for both observations and aggregate analysis. It also systematically overestimated speed, although all speed differences remained within ±3.6 km/h and over 70% within the ±2.0 km/h tolerance as well. Part of these deviations reflects the greater complexity of the curved validation task itself: unlike the tangent section, where the reference mesh allowed continuous spatial comparison, the curved section required users to follow a predefined reference line, introducing variability associated with riding execution.

The results show the value of combining statistical significance with error magnitude, tolerance thresholds, and graphical agreement analysis, following the agreement-analysis logic of Bland and Altman [29]: statistically significant differences were observed in several cases, but their practical relevance depended on whether the errors remained within the predefined tolerances, which are the reference that applies the most for this study.

For the lateral position, the physical scale of the measured phenomena, corresponding to the ±5 cm tolerance defined in Section 2.2.3., which is defined based on the width of the wheel–pavement contact band, is consistent with design practices. The Dutch design guideline [30] defines 20 cm of lateral space for trajectory deviation, established by Godthelp and Wouters (1980) [31]; therefore, the value of ±5 cm corresponds to one quarter of the lateral variability that the infrastructure is itself designed to accommodate. Additionally, this value is 2.3% of a 2.20 m bidirectional bike lane, equivalent to 4.5% of the 1.10 m space available per direction of travel, following the Spanish design guideline recommendations [32]. This level of accuracy is sufficient to characterize users’ lateral position, its variation across control sections in both tangent and curved segments, their position relative to lane boundaries, and the lateral distance between users during meeting or overtaking maneuvers. These variables correspond to the main lateral-position measures commonly used in cycling infrastructure analysis [9,11,12,33].

Complementarily, speed along the alignment in both curved and tangent sections is one of the variables used in the geometric design assessment of bicycle infrastructure [8,9]. For this variable, ±2 km/h represents the finest tolerance at which agreement can be attributed to the tool rather than to the reference, considering the verified deviation of the e-scooter’s speedometer. However, the validation does not support claims regarding instantaneous speed at specific points.

Overall, the validation indicates that the pipeline provides sufficient accuracy to jointly characterize lateral position and speed at specific infrastructure locations (e.g., PC, MP and PT in curved sections, or specific points along a tangent) under controlled experimental conditions.

#### Frame by Frame Comparison

To further validate the estimations obtained by the pipeline, a sub-sample of passing maneuvers was selected for frame-by-frame tracking and measurement. Distance was measured using the front wheel as the reference point, consistent with the current pipeline methodology, and at the same control sections defined above in Figure 5b for the curved section, which represents greater riding task complexity and consequently estimation precision.

Passes were selected using a recursive bisection method: first and last passes, followed by the pass corresponding to the midpoint of the recording and subsequently the midpoints of each resulting interval. This criterion ensured that the sub-sample was distributed throughout the complete videos. Passing maneuvers were matched using the tracker ID, confirming that the same observations were processed by both methods.

Manual tracking was performed using custom code with the same control sections and scale references. No external tool was used in order to avoid additional spatial calibration and maintain methodological consistency. However, operator subjectivity remains a potential source of variability.

For the curved section, a subsample of 114 observations corresponding to 26 passing maneuvers was obtained. At the individual-observation level, the mean lateral distance was 135.77 cm for the manual tracking and 136.39 cm for the pipeline estimates. The resulting mean error was 0.612 cm, with a standard deviation of 5.27 cm, while the MAE and RMSE were 4.13 cm and 5.92 cm, respectively. These results indicate that the pipeline closely reproduces the wheel–pavement contact point identified through manual tracking.

In addition, when the manual tracking results were compared against the 140 cm reference line, the mean error was −4.23 cm, with a standard deviation of 8.29 cm. This differs by less than 1 cm from the −3.52 cm error previously obtained when comparing the full pipeline sample with the reference line. These results suggest that the observed error is mainly related to the users’ trajectories rather than to the estimation performed by the tool. As mentioned above, this may be explained by the greater difficulty of following the reference line through the curve, where users tended to ride slightly inside the line.

Lastly, a reduced but representative subsample was used for this validation because manual tracking is time-consuming and introduces operator subjectivity, particularly in areas where the camera perspective makes the wheel contact point difficult to identify. The average processing time was approximately 11.5 s per observation, equivalent to about 51 min of continuous operator work, although this time may vary depending on the operator. It should also be noted that the manual validation was performed using frames previously identified by the automated pipeline at each control section; for completely manual filtering, identification of the exact moment when each user passes the control section must also be performed manually, and this is the most time-consuming part of the process. In comparison, processing the complete 47 min of video for both sections required approximately 2.5 h with the pipeline.

For larger datasets, the time advantage of the automated pipeline becomes even greater, while also reducing the need for a dedicated operator to work on this task during the manual point selection and data filtering.

### 4.3. Comparison with Existing Methods and Practical Implications

The main practical advantage of the proposed pipeline is that it combines automation with a physically meaningful measurement point. Once the section geometry and control points have been defined, most of the processing workflow runs automatically, reducing manual processing time and enabling the analysis of larger datasets [34,35]. This directly addresses the operator dependency of manual and semi-automatic tools such as Kinovea, used in previous studies [16], in which the measurements rely on the operator’s selection of the reference point and on the consistency of manual tracking [36,37], a source of uncertainty that becomes relevant for bicycles and e-scooters, where the reference point is not always clearly defined.

The second advantage concerns where the measurement is taken. Automated tracking approaches typically locate users through the centroid of the bounding box, which could be adequate for interaction and conflict analysis but does not correspond to any physical point of the vehicle, as it mixes the user’s body, the vehicle, and perspective effects [18,19,23,24]. In contrast, the heatmap-based contour extraction identifies the zone of highest pixel concentration and allows the measurement point to be placed at the contact point between the wheel and the lane surface.

For instance, the centroid of the bounding box was estimated for all analyzed passing maneuvers through the pipeline. For the tangent section, the mean distance obtained using the bounding-box centroid was 186.11 cm, compared with 133.80 cm previously obtained with the pipeline, resulting in a difference of 52.31 cm. The standard deviation of the differences was 7.61 cm, while the MAE and RMSE were 52.31 cm and 52.85 cm, respectively. The minimum and maximum values were 162.19 cm and 203.68 cm, respectively, both substantially higher than those obtained with the pipeline, as shown in Table 3. 

For the curved section, the mean distance obtained using the bounding-box centroid was 180.10 cm, compared with 136.49 cm obtained with the pipeline methodology, resulting in a difference of 43.63 cm. The standard deviation of the differences was 20.23 cm, with an MAE of 43.62 cm and an RMSE of 48.07 cm. The minimum and maximum values were 143.93 cm and 214.70 cm, respectively, also higher than those reported in Table 4.

As further evidence, none of the tangent-section observations estimated using the bounding-box centroid fell within the predefined tolerance limits. For the curved section, only 1.7% of the observations were within ±5 cm, 8.5% within ±10 cm, and 19.3% within ±15 cm, indicating that this approach does not provide representative distance estimates.

Additionally, the location and displacement of the bounding-box centroid do not represent a constant offset that could be removed or corrected through calibration. Since the centroid is determined by the tracker, it can be influenced by rider position, shadows, tracking consistency, and other factors that cannot be reliably estimated or corrected.

For this reason, in micromobility vehicles, which are narrow and single-track, the contact point is precisely what defines the operational position on the infrastructure, making lane positioning and lateral clearance measurements physically interpretable. 

This approach is conceptually close to that of Ronné et al. [25], who estimated the cyclist’s position from the distance between the front-wheel center and a reference line; the present pipeline extends the idea beyond path-following tasks to any delimited section, complementing video methodologies developed for specific layouts such as bike-lane curves [9].

The implementation requirements are also modest. Because the pipeline relies on traditional computer vision, background modeling, and tracking techniques well established for urban traffic video [38,39], it does not require training dedicated models or preparing the annotated datasets on which detection-based approaches typically depend [18,19,23,24,25].

In fact, due to the bird’s-eye-view configuration adopted in this study, existing pretrained datasets were not directly applicable, as they are predominantly based on ground-level images where riders are represented from lateral, frontal, or oblique perspectives, which differ substantially from the top-view perspective used here. For this reason, preliminary tests with a COCO-pretrained YOLOv8 model did not provide reliable detections and therefore could not support the proposed pipeline methodology.

Likewise, the bird’s-eye-view recording configuration is a methodological strength rather than a constraint: it minimizes perspective distortion, simplifies the spatial interpretation of the scene, and is increasingly adopted in field conditions through drone-based acquisition, as reflected in recent naturalistic studies based on aerial imagery [20,35,40]. However, the bird’s-eye-view perspective varies depending on the camera placement and mounting height. Publicly available aerial datasets, such as VisDrone and UAVDT [41,42], contain imagery acquired under different viewing geometries, flight heights, targeted categories, and camera angles and therefore do not necessarily provide representative samples for the close-range top-view configuration adopted in this study.

Lastly, controlled experimental settings are commonly used in micromobility research [11]. Experimental tracks allow researchers to define relevant parameters in advance while maintaining a high level of data validity for operational analyses [43]. Since the tool has been validated under controlled conditions, it is suitable for these types of studies, where the experimental parameters can be established prior to data collection. 

### 4.4. Limitations and Future Research

The validity of the results is bounded by the conditions under which the tool was developed and validated. The current implementation requires bird’s-eye-view recordings, a 30 FPS frame rate, and a 1920 × 1080 camera resolution. The validation was conducted on a controlled section with isolated users, which is the appropriate setting for verifying measurement accuracy against known references; building on this stage, the natural next step is the application of the tool in real-world urban environments, with denser interactions between users and the presence of street furniture. Recording quality also delimits the applicable conditions: the processing parameters are tied to the resolution and frame rate of the input video, and stable camera fixation is required, since wind-induced motion can compromise the continuity of the track maintained by the DeepSORT tracker and, consequently, the consistency of the heatmap from which the contour is extracted. As in most single-camera vision methods, occlusions constrain the analysis, and detection may become less stable at very high speeds, when few frames capture the maneuver, although this is unlikely to affect the intended application, since micromobility speeds in controlled infrastructure studies generally remain below 25 km/h [44].

These limitations define the main lines of future development. The treatment of shadows can be strengthened: Since the contour extraction is based on a pixel concentration threshold derived from the accumulation profile, and the user typically generates a higher concentration than the projected shadow, the filtering parameters can currently be adjusted case by case, or, furthermore, another methodology that includes a robust cast-shadow removal prior to contour extraction could be developed [45,46,47,48]. Extending the tool to oblique and wide-angle recordings involves incorporating standard geometric processing, namely camera calibration, lens-distortion correction, homography matrices, and the transformation of image coordinates into real-world coordinates [49]. Currently, the pipeline has only been tested under outdoor experimental conditions and other outdoor controlled scenarios. Future work should therefore validate its performance under real traffic conditions and exploit its multi-user tracking capability for interaction and conflict analysis, as well as for the automatic classification of maneuver types. In addition, the images collected during these tests could be used to progressively build a dedicated dataset [18,19,22], covering representative camera configurations and heights commonly used in micromobility studies for safety assessment.

## 5. Conclusions

This study developed and validated a semi-automated computer-vision tool for extracting the instantaneous speed and lateral position of micromobility users from bird’s-eye-view video recordings. The tool, implemented in Python 3.10.11, is structured into four processing stages: bike lane segmentation, user detection and tracking, path contour extraction, and quantification of lateral position and speed at predefined control points.

A key contribution of the method is the use of heatmap-based contour extraction to estimate the user’s operational trajectory. This allows the measurement point to be placed at the wheel–pavement contact of bicycles and e-scooters, rather than relying on the centroid of the bounding box, which is particularly relevant for micromobility studies, where small variations in lateral position affect the interpretation of lane use and the interaction with infrastructure elements.

The validation, conducted in tangent and curved sections against physical distance references and previously verified e-scooter speed readings, combined error metrics, inferential tests, agreement analyses and tolerance thresholds, defined based on criteria that represent the evaluated phenomena.

The tool was validated for both variables in tangent and curved sections. In the tangent, lateral position showed negligible bias, with over 95% of measurements meeting the ±5.0 cm criterion, while speed was slightly underestimated, with more than 70% within ±2.0 km/h tolerance. In the curve, lateral position was underestimated, with 41.7% meeting the ±5.0 cm criterion, whereas speed was slightly overestimated, with nearly 80% within a ±2.0 km/h limit. These differences are mainly attributed to natural riding task complexity in the curved section, as confirmed by the frame-by-frame analysis. The results also support the wheel–pavement contact point as the positional reference, since centroid-based estimates poorly represented the rider’s actual ground position from a bird’s-eye view where only 1.7% of the observations fell within the ±5.0 cm criterion in the curve, and none of them did so in the tangent.

Overall, the validation supports the use of the tool for operational and safety studies of micromobility in experiments under controlled conditions. Because it relies on traditional computer vision rather than supervised detection models, it does not require training dedicated models or preparing annotated datasets, which simplifies its implementation. Its semi-automated structure reduces operator subjectivity and substantially decreases processing time compared with manual or other semi-automatic video analysis, and it generates complete outputs (processed videos, heatmaps, trajectories, visual overlays, and structured data files) that can support engineering applications by identifying where users circulate within bicycle facilities and by jointly analyzing speed and lateral position as complementary indicators of riding behavior. In addition, since the tracking stage can handle more than one user in the same scene, the tool provides a basis for future interaction and conflict analyses.

Future work will extend the method to oblique and wide-angle perspectives through camera calibration and homography transformation, include shadow-removal procedures, and validate the tool under real-world traffic conditions in more diverse micromobility scenarios.

## Figures and Tables

**Table 1:** Error metrics by speed.

Speed	*n*	Mean	SD	MAE	RMSE	Max. Absolute Error
10	10	0.263	0.253	0.282	0.356	0.607
15	10	−0.024	0.228	0.191	0.218	0.378
20	10	−0.566	0.509	0.598	0.744	1.405
Total	30	−0.109	0.489	0.357	0.493	1.405

**Table 2:** Tolerance-based assessment for speed measurements.

Speed (km/h)	Tolerance (km/h)		
	±0.2 (%)	±0.5 (%)	±1.0 (%)
10	6/10 (60)	7/10 (70)	10/10 (100)
15	4/10 (40)	10/10 (100)	10/10 (100)
20	3/10 (30)	5/10 (50)	9/10 (90)
Total	13/30 (43.3)	22/30 (73.3)	29/30 (96.7)

**Table 3:** Descriptive metrics for lateral position validation in the tangent section.

Method	Mean (cm)	Median (cm)	SD (cm)	Min (cm)	Max (cm)
Ground truth	133.65	135.00	8.23	110.00	155.00
Pipeline	133.79	134.66	8.05	106.50	157.11
Error	0.14	0.24	2.58	−11.27	6.89

**Table 4:** Descriptive metrics for lateral position validation in the curve section.

Method	Mean (cm)	Median (cm)	SD (cm)	Min (cm)	Max (cm)
Ground truth	140	-	-	-	-
Pipeline	136.49	136.46	7.42	121.86	150.86
Error	−3.51	−3.54	7.42	−18.14	10.86

**Table 5:** Aggregated descriptive metrics for lateral position validation in the tangent section.

Method	Mean (cm)	Median (cm)	SD (cm)	Min (cm)	Max (cm)
Ground truth	133.65	135.00	6.10	122.00	146.60
Pipeline	133.80	134.22	6.28	122.10	146.45
Error	0.14	0.26	1.380	−3.55	2.35

**Table 6:** Aggregated descriptive metrics for lateral position validation in the curve section.

Method	Mean (cm)	Median (cm)	SD (cm)	Min (cm)	Max (cm)
Ground truth	140.00	140.00	-	-	-
Pipeline	136.24	135.43	4.76	126.48	145.31
Error	−3.76	−4.57	4.76	−13.51	5.31

**Table 7:** Descriptive metrics for speed validation in the tangent section.

Method	Mean (km/h)	Median (km/h)	SD (km/h)	Min (km/h)	Max (km/h)
E-scooter	11.96	11.5	3.20	7.50	19.00
Pipeline	11.46	10.74	3.23	7.31	18.67
Error	−0.49	−0.39	1.50	−3.02	2.14

**Table 8:** Descriptive metrics for speed validation in the curve section.

Method	Mean (km/h)	Median (km/h)	SD (km/h)	Min (km/h)	Max (km/h)
E-scooter	8.31	7.50	0.99	7.00	10.50
Pipeline	9.63	9.75	1.13	7.54	11.77
Error	1.32	1.27	0.76	0.02	2.948

**Table 9:** Summary of validation performance. * Tolerance = ±5 cm. ** Tolerance = ±2.0 km/h.

Variable	Section	*n*	Mean Bias	MAE	RMSE	Within Main Tolerance
Lateral position *	Tangent	140	+0.14 cm	2.02 cm	2.57 cm	95.7%
		28	+0.14 cm	1.11 cm	1.36 cm	100%
	Curve	295	−3.51 cm	6.77 cm	8.20 cm	41.7%
		50	−3.76 cm	5.18 cm	6.03 cm	50%
Speed **	Tangent	26	−0.49 km/h	1.26 km/h	1.55 km/h	73.1%
	Curve	69	+1.33 km/h	1.33 km/h	1.53 km/h	79.7%

## Data Availability

The source code of the tool is openly available on GitHub at https://github.com/alfoncab/micromobility-cv-tool since 13 July 2026 and permanently archived in Zenodo at https://doi.org/10.5281/zenodo.21334112 since 13 July 2026. The validation datasets presented in this article are available on request from the corresponding author.

## Abbreviations

The following abbreviations are used in this manuscript:

IMU	Inertial Measurement Unit
RoI	Region of Interest
PC	Point of curvature
MP	Midpoint
PT	Point of tangency
INT	Intermediate points
